# A Highly Productive, Whole-Cell DERA Chemoenzymatic Process for Production of Key Lactonized Side-Chain Intermediates in Statin Synthesis

**DOI:** 10.1371/journal.pone.0062250

**Published:** 2013-05-07

**Authors:** Matej Ošlaj, Jérôme Cluzeau, Damir Orkić, Gregor Kopitar, Peter Mrak, Zdenko Časar

**Affiliations:** 1 Genetics, Anti-Infectives, Lek Pharmaceuticals d.d., Mengeš, Slovenia; 2 API Development, Sandoz Development Center Slovenia, Lek Pharmaceuticals d.d., Mengeš, Slovenia; 3 Faculty of Pharmacy, University of Ljubljana, Ljubljana, Slovenia; Queen’s University Belfast, United Kingdom

## Abstract

Employing DERA (2-deoxyribose-5-phosphate aldolase), we developed the first whole-cell biotransformation process for production of chiral lactol intermediates useful for synthesis of optically pure super-statins such as rosuvastatin and pitavastatin. Herein, we report the development of a fed-batch, high-density fermentation with *Escherichia coli* BL21 (DE3) overexpressing the native *E. coli deoC* gene. High activity of this biomass allows direct utilization of the fermentation broth as a whole-cell DERA biocatalyst. We further show a highly productive bioconversion processes with this biocatalyst for conversion of 2-substituted acetaldehydes to the corresponding lactols. The process is evaluated in detail for conversion of acetyloxy-acetaldehyde with the first insight into the dynamics of reaction intermediates, side products and enzyme activity, allowing optimization of the feeding strategy of the aldehyde substrates for improved productivities, yields and purities. The resulting process for production of ((2*S*,4*R*)-4,6-dihydroxytetrahydro-2*H*-pyran-2-yl)methyl acetate (acetyloxymethylene-lactol) has a volumetric productivity exceeding 40 g L^−1^ h^−1^ (up to 50 g L^−1^ h^−1^) with >80% yield and >80% chromatographic purity with titers reaching 100 g L^−1^. Stereochemical selectivity of DERA allows excellent enantiomeric purities (*ee* >99.9%), which were demonstrated on downstream advanced intermediates. The presented process is highly cost effective and environmentally friendly. To our knowledge, this is the first asymmetric aldol condensation process achieved with whole-cell DERA catalysis and it simplifies and extends previously developed DERA-catalyzed approaches based on the isolated enzyme. Finally, applicability of the presented process is demonstrated by efficient preparation of a key lactol precursor, which fits directly into the lactone pathway to optically pure super-statins.

## Introduction

Statins, inhibitors of 3-hydroxy-3-methylglutaryl-coenzyme A (HMGCoA) reductase [1]–[3], are one of the most pronounced success stories of modern medicinal chemistry [4]. Beside their cholesterol-lowering capabilities, they have been found also to possess many other beneficial effects [5]–[9]. Statins consist of a chiral diol side chain, appended to a cyclic fragment. Initially discovered as microbial metabolites [10]–[13], statins have been rapidly developed into even more efficient synthetic analogues by a partial modification of their structure. The fully synthetic derivatives are frequently addressed as super-statins [14]. Due to the fact that the side chain represents an essential building block in all statins, vast research efforts have been made for its efficient construction and incorporation into the final statin structure. These studies have demonstrated that statins are readily accessible through a variety of different approaches utilizing various types of side-chain precursors. Indeed, statins have been built from open side-chain derivatives, lactol type precursors [14] and very recently from a lactonized side-chain derivative [15], [16]. Due to the increased commercial demand for statins and the need for a simple preparation on industrial scale, impetus for even more efficient, environmentally friendly and easily scalable preparation of the side-chain derivatives appeared. Moreover, synthetically demanding chiral diol structure of the side chain and high quality requirements of pharmaceutical industry on its (stereo)chemical purity shifted the research from initially pure chemical synthesis to enzyme-based approaches, which are known to perform with high stereoselectivity [17]–[32].

**Figure 1:**
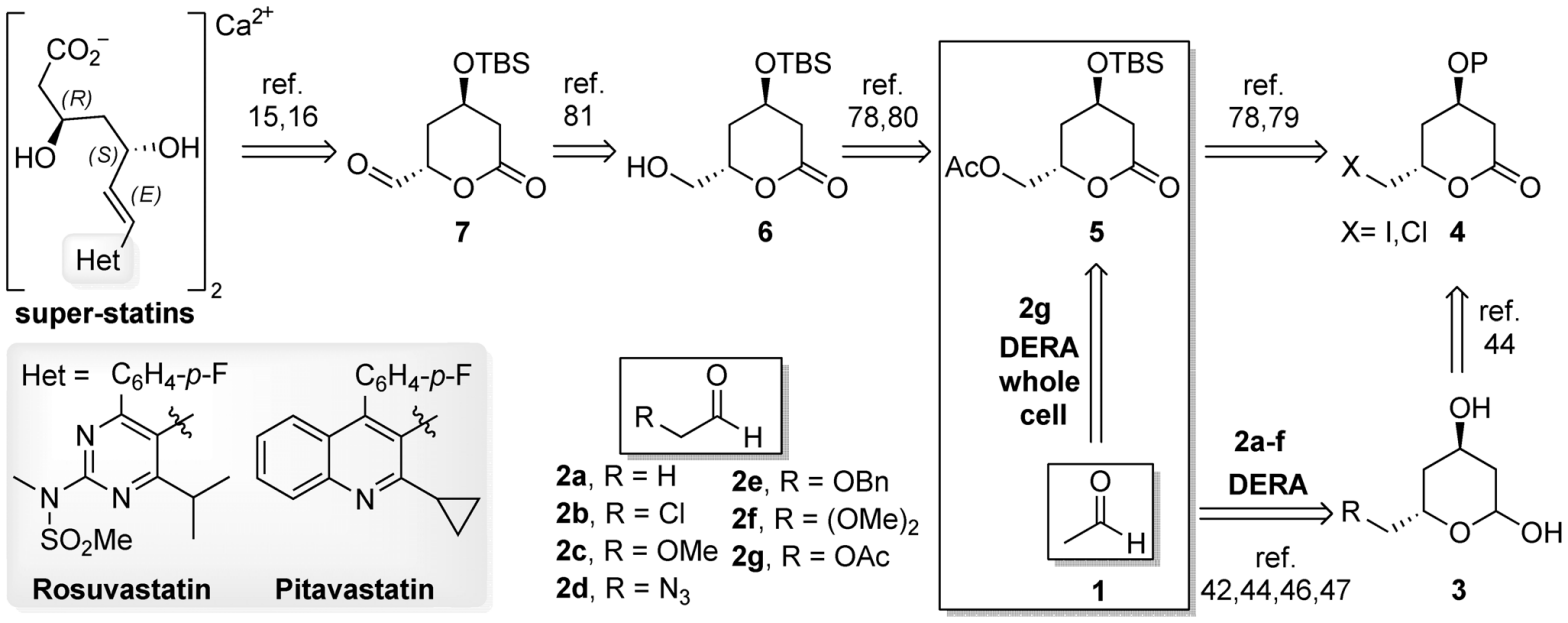
Current art in the direct synthesis of optically pure super-statins from the lactol precursors.

Among applications of various enzymes in synthesis of the statin side chain [33]–[36], utilization of 2-deoxyribose-5-phosphate aldolase (DERA) [37]–[41] for the synthesis of chiral lactol precursors [42]–[47] attracted considerable attention recently. This is due to the fact that DERA-catalyzed aldol condensations [48]–[51] of prochiral aldehyde precursors **(1)** and **(2)** provide lactols **(3)** (Figure 1), containing two chiral centers of the final statin side chain with excellent stereopurity and high yield. DERA (EC 4.1.2.4) is a lysine-mediated aldolase (type I), able to accept acetaldehyde **1** (donor) and glyceraldehyde-3-phosphate (acceptor) as its natural substrates [52]–[55]. This ubiquitous enzyme is unique among aldolases by being capable of condensing two aldehydes and is well-known for its relaxed substrate specificity; both on the donor and the acceptor side [42]–[43], [56]–[58]. While acetaldehyde **1**, acetone, flouroacetone and propanal can be used as donors, even broader selection is possible for acceptor molecules. In addition to a remarkable number of various aldehydes, aldose-sugars and their phosphates can also be accepted [42], [56]–[58]. Use of DERA for *in vitro* catalysis with unnatural substrates, involved in single DERA-catalyzed aldol condensation, was first shown by Barbas *et al.* [56], followed by description of sequential aldol condensations catalyzed by *E. coli* DERA [42], [57], which opened a new field in chemoenzymatic synthesis. Several 6′-substituted dideoxy-sugars can be prepared using DERA in high enantiomeric purity [42], [57]. An immediate application of this reaction was reported by Greenberg *et al.* [44], using an alternative DERA enzyme in synthesis of chiral (halo)lactols, where *ee* of >99.9% and *de* >96.6% are reported in a highly productive and scalable process for production of (4*R*,6*S*)-6-(chloromethyl)tetrahydro-2*H*-pyran-2,4-diol (**3b**). Other processes employing DERA for sequential aldol condensations have been reported since [46], [59]–[60].

These processes, however, have some major drawbacks. One of them, arising from a high enzyme inactivation rate (driven by the aldehyde-lysine coupling), is the high catalyst load necessary for reactions with industrially suitable yields and productivities [61]. Apart from interfering with surface lysine residues (thereby destabilizing the enzyme’s tertiary structure) acetaldehyde (**1**) has a detrimental effect on the enzyme’s activity when the catalytic lysine residue in the active site is inactivated [38], [61]–[62].

The problem of DERA inactivation in the presence of aldehydes has been addressed before either by employing random (epPCR, DNA shuffling) and/or targeted mutagenesis [61], [63] or, alternatively, by screening of environmental DNA libraries [44] and immobilization procedures [64]–[66]. The immobilization approach aimed at improving stability of DERA was attempted also in our laboratory (unpublished data), however this approach was found to be economically unfavorable. While great efforts have been invested into resolving the inactivation problems in DERA-catalyzed reactions, a high enzyme load is still necessary for high-titer processes, making the biocatalyst a major cost driver. In contrast to the described techniques, which stream toward reduction of the enzyme load or its recycling, a different approach, aiming at reduction of the price impact of the biocatalyst, is to minimize its production costs.

Compared to even the most straightforward enzyme downstream procedures, the use of a whole-cell biocatalyst [67]–[73] in form of a fermentation broth is by far the most inexpensive approach to biocatalysis unless very stable and recyclable enzymes can be used [73]. Several industrially scalable whole-cell bioconversion processes utilizing *Escherichia coli* as the enzyme expression host are known today [67]–[77]. The feasibility of such approach, however, is limited by specific properties of each individual case of enzyme and bioconversion process. One major prerequisite for a whole-cell catalyzed process, for example, is the permeability of cellular envelopes for substrates and products when considering a cytoplasmic expression of the enzyme. Another important prerequisite is the absence of secondary enzymatic activities in the cell biomass, which could lower the yields of the desired product by further metabolization (e.g. cleavage by hydrolyses in the case of **2g,** see the results section) or by catabolic utilization of the reaction substrates. Third, but equally important criterion is the absence of impurities either derived from the fermentation medium or produced by the host microorganism, which would influence overall product quality and result in additional, undesired purification steps.

Although several implications for suitability of (halo)lactols **(3)** obtained *via* DERA-catalyzed approaches in statin synthesis have been made, a clear-cut direct application of these intermediates for assembly of statins with heptenoic side-chain residue has remained vague. This is due to the fact that usually lactols **(3)** need to be first oxidized to lactones **(4)**, then ring-opened and after several synthetic steps (including protection/deprotection sequences) side-chain derivatives suitable for super-statin assembly are obtained [44]. All these additional chemical steps in the total synthesis devalorize highly effective and straight-forward enzymatic step. Recently, we have developed the most efficient chemical approach to halolactone derivative **(4)** [78]–[79], which has been successfully hydroxylated *via* S_N_2 reaction with acetates to acetyloxymethylene-lactone **(5)** followed by deacetylation with tin catalyst [78], which was latter replaced for a nontoxic, environmentally acceptable and inexpensive chemoselective acetyl cleavage [80], catalyzed by the pancreatic lipase powder to give **(6)**. Furthermore, the obtained hydroxymethylene-lactone **(6)** was for the first time successfully transformed to its formylated analogue **(7)** suitable for coupling with the heterocyclic counterpart [81]. Moreover, we have demonstrated for the first time that statins can be directly assembled from the lactonized side-chain precursor **(7)** [15], [16].

**Figure 2:**
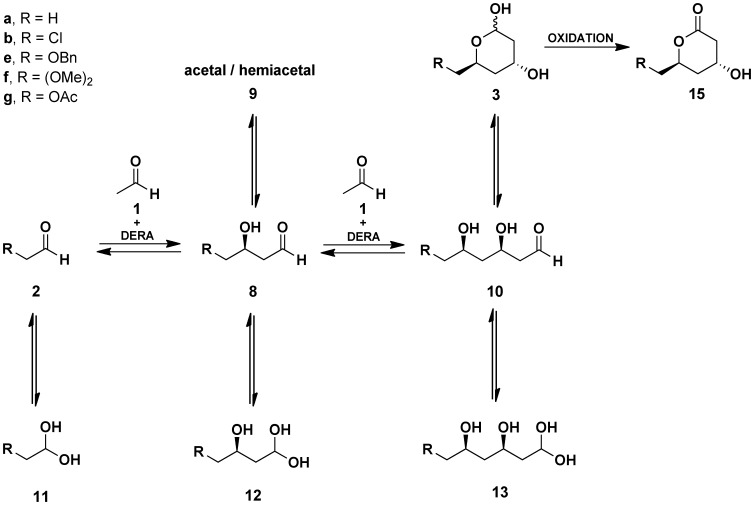
The DERA-catalyzed sequential aldol condensation with 2-substituted acetaldehydes. Several acetal/hemiacetal (**9**) species were found in equilibrium with **8**. These may include monomeric cyclic hemiacetal as well as dimeric and trimeric cyclic acetals/hemiacetals (Information S3). Compounds **15** are obtained by chemical oxidation of the lactols **3**.

Having proved the direct lactone pathway to statins, we have opened a further possibility for direct application lactols **(3)**/lactones **(4)**/**(5)** derived *via* DERA-catalyzed approach without the necessity of their lengthy transformation to dihydroxyl protected open-chain derivatives before the final statin formation. Therefore, we were prompted to prepare a key acetyloxymethylene-lactone **(5)** precursor *via* DERA-catalyzed approach (Figure 2) from acetaldehyde **(1)** [82] and aldehyde **(2g)** [83] in order to take advantage of its efficient assembly into statin structure and thus provide efficient total synthesis of super-statins based on enzymatic technology (Figure 1) [84].

Here, we report development of a high-density *E. coli* fermentation process, yielding a broth with high activity of DERA. By directly utilizing this broth in a highly productive, high-yield, whole-cell, chemo-enzymatic process, we show that all of the above outlined challenges can be successfully overcome. Special focus is given to a process yielding acetyloxymethylene-lactol **3g** wherein we point out the role of reaction kinetics of the two sequential condensation steps for the outcome of the reaction. Measurement of the reaction intermediate (**8**) and side-product (**3a**) accumulation allowed optimization of the feeding strategy, which proved to be a yield-determining parameter. Further conversion to advanced downstream statin intermediates confirms the efficient total synthesis of super-statins based on enzymatic technology and employing our previously described methodology [15]–[16], [78]–[81], [83].

## Materials and Methods

### Strains and Culture Conditions

*E. coli* BL21 (DE3) (Invitrogen, USA) was used for the expression of wild type *E. coli* DERA from the pET30a+plasmid (Novagen, USA). The expression plasmid was constructed by PCR amplification of the *deoC* gene from *E. coli* DH5α genome using primers GCCGATATCCGTAGCTGCTGGCGCTCTTACC and CGGCATATGACTGATCTGAAAGCA-AGCAGCC followed by cleavage of the amplified fragment as well as the host plasmid with *Nde*I and *Blp*I restriction endonucleases and assembly of the fragments in a T4 ligation reaction to yield pET30/*deoC*. All shake flask cultivations were performed on a rotary shaker (250 rpm, 5 cm radius) at 28°C. The seed medium (VD) consisted of Yeast extract (10 g L^−1^), NaCl (5 g L^−1^), glycerol (5 g L^−1^) and NaH_2_PO_4_ (2 g L^−1^) and was supplemented with Kanamycin (25 µg mL^−1^). The seed culture was prepared by inoculation of 30 mL of fresh VD medium (250 mL shake flask) with 200 µL of overnight *E. coli* BL21 (DE3) pET30/*deoC* VD culture. After approximately 8 h of cultivation at 28°C, OD_600_ reached 1.5–2.0 and the seed culture was used for inoculation of the main culture. Expression of DERA in a high-density fermentation culture was performed in a 2 L bioreactor (ISF-100, Infors HT, CH) The main fermentation medium and the glucose feeding solution were described before [85] and were both supplemented with kanamycin (25 µg mL^−1^). 1 L of the main medium was inoculated with 15 mL of seed culture and was grown with initial conditions (28°C, pH 7.0, 700 rpm, 1 L min^−1^ aeration, atmospheric pressure) until dO_2_ dropped to 30%. After that, rpm and airflow were automatically raised in order to maintain the dO_2_ above 30% (1800 rpm and 3 L/min were the maximum settings reached). pH was adjusted automatically to 6.8, using ammonia solution (12.5%). The feeding with glucose solution was initiated upon depletion of the glycerol in the main fermentation medium which was indicated by an instant drop in oxygen consumption (10–12 h into the process). The flow rate for the feeding solution was initially set to 0.08 mL min^−1^ and exponentially increased to 0.27 mL min^−1^ in the course of 24 h. The expression was induced by addition of 0.2 mM IPTG 18 h after inoculation. The resulting culture broth (DERA fermentation broth) was harvested between 34 and 39 h after inoculation and used directly in the DERA reactions. Alternatively, cell-free lysates were used (where indicated), prepared by sonication of the culture broth (Digital Sonifier 450, Branson, USA; std. horn, T <8°C, 5×10 s pulses, 70% amplitude), followed by centrifugation in order to remove cell debris.

### DERA Activity Measurements

Activity of DERA was assayed using 7-deoxyribosyl-4-methyl umbelliferone as reported by Greenberg *et al.* [44], [60], [63]. Fluorescence was measured for 30 min at 28°C using fluorescence spectrometer SpectraMax M2 (Molecular Devices, USA) set to excite at 370 nm and detect emission at 455 nm. The following modifications to the original method were made. 96-well micro-titer plates (96w Costar blk/blk bottom), covered with an optically transparent foil (MicroAmp, Applied Biosystems, USA) were used for the measurements. Samples were diluted 1000 fold in buffer (100 mM Bis-Tris propane, pH 8.5, total volume 200 µL). Each of the 7 different loads of properly diluted sample, spanning from 10 µL to 170 µL (2 µg to 40 µg of biomass per assay; OD_600_: 0.005 to 0.1), was measured in triplicate after addition of 10 µL of BSA (40 mg/mL) and 20 µL of the fluorogenic substrate [44] (∼ 1.5 mg/mL in 20% DMSO; corrected by dilution to match A_320_ = 65.0±0.5 at 28°C) for determination of reaction velocity. Wet cell weight (WCW; weight of sediment per sample volume after centrifugation at 16000 G for 10 min) of samples was measured prior to the activity measurement and was used to calculate specific activity of the biomass. In cases where cell-free lysates were used as catalysts, the WCW of the source culture was used for specific activity calculation for practical reasons. The specific DERA activity was determined as slope of the mean fit to at least 5 points in an initial velocity vs. biomass load plot and is given in kRFU s^−1 ^g^−1^ as an average of the triplicate samples. Zero value for blank sample was included, but not forced in the linear regression. Acceptance criteria for a valid measurement were: R^2^>0.99, offset <0.02.

### General Reaction Conditions

The substrates acetaldehyde **1**, chloracetaldehyde **2b**, dimetoxyacetaldehyde **2f** and benzyloxyacetaldehyde **2e** used for the reaction were obtained from Sigma and were of p.a. grade. Acetyloxyacetaldehyde **2g** was prepared as described before [83]. All reactions were performed in a 2 L stirred vessel (ISF-100, Infors HT, CH) at 37°C. pH was adjusted automatically using ammonia solution (12.5%) to 6.2. Stirrer speed was set to 700 rpm. The *E. coli* BL21 (DE3) pET30/*deoC* fermentation broth was added first (80% of the final volume of the reaction). Substrates were added directly into the reaction mixture into the vicinity of Rushton impeller using a programmable pump (Costametric 4100, Thermo separation products, US) with the acetyldehyde being diluted in water so that both substrates filled the remaining 20% of the final reaction volume. The condenser of the reactor exhaust was cooled to 2°C in order to minimize the loss of acetaldehyde **1** due to evaporation.

### GC-MS and GC-FID Analytics

Reaction-mixture samples were quenched with 4 volumes of acetonitrile. Cells and precipitates were removed by centrifugation and the samples were further diluted by a factor of 10 in acetonitrile. Analyses were performed on an Agilent (USA) 7890A gas chromatographic (GC) system equipped with flame ionization detector (FID). A HP-5MS column with dimensions of 60 m×250 µm (ID) ×0.25 µm was used. 1 µL of samples was injected onto the column by split injection (split ratio 20∶1) via inlet, which was held at 200°C. Helium (6.0) was used as a carrier gas at constant pressure of 45 PSI (initial flow 3.4 mL min^−1^). Temperature program was set as follows: initial temperature 50°C (5 min), gradient 10°C min^−1^ until 250°C (5 min). Analytes were detected on a FID detector. Detector temperature was set to 250°C. Gas flow rates were set to 30 mL min^−1^ for hydrogen gas (fuel), 300 mL min^−1^ for air (oxidizer) and 25 mL min^−1^ for nitrogen gas (make-up).

For the GC-MS analytics, samples were prepared as above and injected into GC-MS system with chemical ionization (CI) for determination of CI mass spectrum. Analysis were performed on an Agilent 7890A gas chromatographic (GC) system coupled with Agilent mass selective detector 5975C inert XL EI/CI MSD using inert chemical ionization (CI) source. Methane gas was used as a chemical ionization reagent to perform a positive chemical ionization (PCI). 1 µL of sample was injected onto a HP-5 Trace Analysis 5% Phenyl column with dimensions of 30 m×250 µm (ID) ×0.25 µm by split injection (split ratio 20∶1) via inlet, which was held at 200°C. Helium (6.0) was used as the carrier gas at constant pressure of 10 PSI (initial flow 1.2 mL min^−1^). Temperature program was set as follows: initial temperature 50°C (5 min), gradient 10°C min^−1^ until 230°C (5 min).

### Isolation of the Reaction Products and Intermediates

After the completion of reactions, pH was lowered to 4.5 using phosphoric acid. 200 g L^−1^ of Na_2_SO_4_ was added in order to facilitate extraction of reaction products and intermediates with EtOAc. Whole-broth extraction with 2 vol. of EtOAc was repeated 4 times. EtOAc phase was collected, dried over MgSO_4_ and filtered, followed by evaporation yielding a crude product in the form of a yellow-brownish oil. Where pure compounds were needed, the oil was dissolved in water and mixed with 5 vol. of toluene, which was then removed and discarded. Aqueous phase was extracted with 2 vol. of CH_2_Cl_2_. The first extract was discarded and the extraction was then repeated 6 times. The CH_2_Cl_2_ phase was collected, dried over MgSO_4_ and evaporated. The resulting light yellow oil was dissolved in 95/5 water/acetonitrile (500 g L^−1^) and injected (1 mL) into a reverse phase preparative LC system (Äkta Purifier, Amersham-Biotech, SE) using the preparative C18 column (EVF D17, RP18 25–40 um −15 g, Merck Chimie SAS). Flow rate of 2.5 mL min^−1^ was used in isocratic mode with 95/5 water/acetonitrile (pH = 5) for the first 55 min, followed by a gradient from 95/5 to 50/50 (water/acetonitrile) over the next 15 min. The resulting 2 mL fractions were analyzed by GC-FID and the fractions containing the desired compound were collected. Acetonitrile was evaporated and the compound was extracted from the aqueous phase with CH_2_Cl_2_ to give pure compounds.

### Chemical Oxidation of the Lactols

To a whole-cell catalyst reaction mixture with **3g** (250 mL, **3g** concentration 55.0 g/L), NaCl (83.9 g, 1456 mol) was added at r.t. and mixed until dissolved using mechanical stirring. Then, EtOAc was added (83 mL) and cooled to 0–5°C, pH adjusted to 3 by phosphoric acid and to this solution, Ca(OCl)_2_ (65%, 37.7 g, 171.3 mmol) was added portion-wise over 3 h, keeping the pH between 2–6 (by phosphoric acid) and the temperature range between 5–25°C by cooling with ice bath. After additional 0.5 h, the reaction was finished and chlorine was driven out by bubbling N_2_ through the solution (exhaust going to a trap consisting of solution of NaOH and Na_2_S_2_O_3_ in water). From the reaction mixture, EtOAc was distilled under reduced pressure, Celite® (10 g) was added and the mixture filtered. The filter cake was washed with 2×80 mL of distilled water and combined filtrate fractions were extracted with CH_2_Cl_2_ (10×250 mL), The CH_2_Cl_2_ phases were dried with CaCl_2_, filtered and evaporated to obtain **15g** (14.6 g, 85.5% assay, 92% crude yield).

## Results and Discussion

### High-density Fed-batch Fermentation

The fermentation process for production of DERA whole-cell biocatalyst was developed with three targets in mind. First, the volumetric DERA activity should support direct use of the fermentation broth for a highly productive, chemoenzymatic process comparable to the process described by Greenberg *et al.* [44]. Secondly, the use of a defined mineral medium with means of controlling the residual carbon source would minimize contaminating impurities during the work-up of the chemoenzymatic reaction broth. And thirdly, the raw-material and process costs should be as low as possible in order to make the process not only industrially scalable but also economically favorable compared to any other DERA-catalyzed process known. The process outline was based on a mineral-medium, high-density *Escherichia coli* fermentation protocol [85], which, however, is focused on biomass production and not specifically evaluated for protein expression. We have found, nevertheless, that the protocol allows appropriate high-density process for a high-level DERA expression when the time of induction, inducer concentration and carbon source feeding rates are optimized (Information S1). Typical characteristics of the final broth with the optimized procedure, harvested at 36 h after inoculation were: 180–215 g L^−1^ wet cell weight (OD_600_ ∼ 100–110), 210–250 kRFU s^−1 ^g^−1^ specific DERA activity. The DERA protein was estimated to be present in 4.07 g L^−1^ and represented ∼ 50% of the total soluble protein after analysis of cell-free extracts with SDS-PAGE (Information S1). In comparison, shake-flask experiments using standard expression conditions (LB, 37°C, 1 mM IPTG at OD_600_ = 0.6, 6 h induction time) yielded 8 g L^−1^ WCW and 300 kRFU s^−1 ^g^−1^.

The resulting culture broth (DERA whole-cell catalyst) was used directly in the aldol condensation reactions. Raw material cost calculation (using bulk prices of raw material) for this process, which was successfully scaled-up to 70 L and 200 L bioreactors in a linear manner from the 2 L scale protocol, was calculated to be less than 0.2 € per kg of the whole-cell catalyst. According to the advances in high-density *E. coli* fermentation, [86], [87] given additional optimization, it should be possible to increase the biomass yields, and consequently, the volumetric DERA activity of the fermentation broth even further.

### Activity Assay

**Figure 3:**
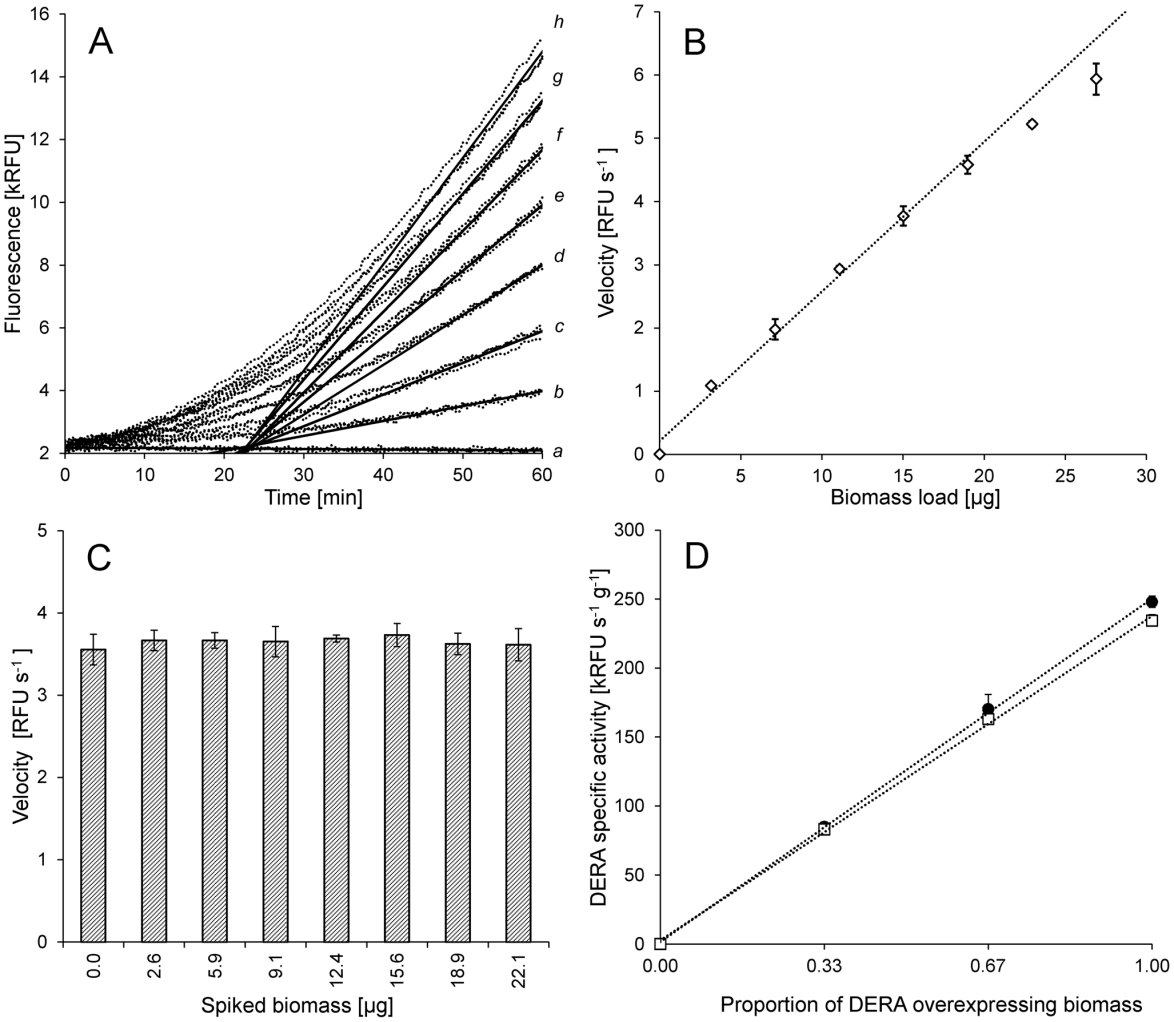
DERA activity measurements of the whole-cell catalyst. **A.** Fluorescence raw data for a DERA activity assay (dotted lines). Velocities for triplicate samples of the whole-cell catalyst were measured for 7 different loads (*b–h,* 3.16 µg–26.9 µg in 3.96 µg increments) of biomass. After normalization with the blank (*a*), maximum slopes were determined for each sample and averaged (solid lines) to yield velocity for a given biomass load. **B.** Velocity vs. biomass load plot. The first 5 points are taken for the specific activity calculation. Linear regression: y = 0.2366x+0.2073 R^2^ = 0.9936 **C.** Comparison of velocities measured for cell-free lysate spiked with increasing loads of biomass. **D.** Validation of linearity of the activity assay within samples with constant biomass. The whole-cell catalyst *E. coli* BL21 (DE3) pET30/*deoC* was mixed with w.t. *E. coli* BL21 (DE3) biomass (•). Linear regression: y = 248.94x+1.3840, R^2^ = 0.9995. In parallel, sonicated and cleared samples were measured (□). Linear regression: y = 235.00x+2.6433, R^2^ = 0.9989.

Due to the well-known inactivation of DERA by the aldehyde substrates [38], [61]–[62], and the fact that a whole-cell catalyst was to be used for chemoenzymatic reactions, a straightforward and accurate activity assay to support the work was essential and therefore given much attention. The fluorometric method for determination of DERA activity was described before [44], [60], [63], however it’s precision on a whole-cell catalyst remained to be tested. A series of experiments was performed with the aim to evaluate the impact of cellular envelopes on the efficiency of the retro-aldol reaction resulting in release of the fluorescent 4-methyl umbelliferone from the relatively bulky substrate 7-deoxyribosyl-4-methyl umbelliferone the cell-free extracts were always compared to the whole-cell catalyst from which they originated). In addition, the light scattering due to the presence of the cells in the samples may have an impact on the fluorescence readings [88]. The latter was evaluated by measuring activity of a cleared DERA lysate spiked with increasing amounts of DERA-expressing cells in the range between 0.01 and 0.12 g L^−1^ WCW (OD_600_ = 0.005 to 0.012). In this range, independent measurements showed high linearity (R^2^>0.99) in velocity vs. biomass-load plot for the appropriately diluted samples of the DERA fermentation broth (Figure 3a and 3b). The spiking of the cell-free lysate had no effect in the range described (Figure 3c). Linearity of specific biomass activity within samples with constant biomass concentration was validated using mixtures of *E. coli* BL21 pET30/*deoC* cultures with high DERA activity and w.t. *E. coli* BL21 cultures. The cell concentration in the samples was kept constant in the aforementioned range, within which, the fraction of the DERA-expressing cells varied. In parallel, measurements were performed with cell-free extracts of identical samples, prepared by thorough sonication and removal of cell debris by centrifugation (Figure 3d). The results show highly linear response both for the whole-cell catalyst and the cell-free extract mixtures. Moreover, in comparison, both catalysts had specific activities at essentially the same level. Data obtained in a similar experiment, where the cell-free lysate was prepared directly from the high-density DERA fermentation broth, and activity of the two was compared after appropriate dilution, were in agreement with the above.

To confirm that the activity detected on the whole cells is not due to potential lysis of the cells and release of DERA extracellularly during the fermentation or the activity assay, the following measurements were made. First, the supernatant of a freshly harvested high-density culture of *E. coli* BL21 pET30/*deoC* was analyzed. The activity of the extracellular DERA contributed 4.5±2% to the total fermentation broth activity, whereas the washed biomass contributed the remainder of the activity (Information S6). Next, using SDS-PAGE, we compared the amount of extracellular enzyme present in the assay mixture at the end of the measurement where either the cleared lysate or the whole broth was used as a catalyst. The results clearly show that lysis of the cells during the assay is minimal, since only traces of the DERA protein were found in the whole-cell assay supernatant compared to the cleared-lysate assay mixture. At the same time, similar activity values were measured for both samples (Information S6). Taken together, the results strongly suggest that the bulky 7-deoxyribosyl-4-methyl umbelliferone is used as a substrate by the intracellularly expressed DERA and that both the substrate and the product, 4-methyl umbelliferone, freely diffuse in and out of the cells during the assay. Whether this is a simple diffusion process or an assisted transport mechanism is involved, remains beyond our investigations.

**Figure 4:**
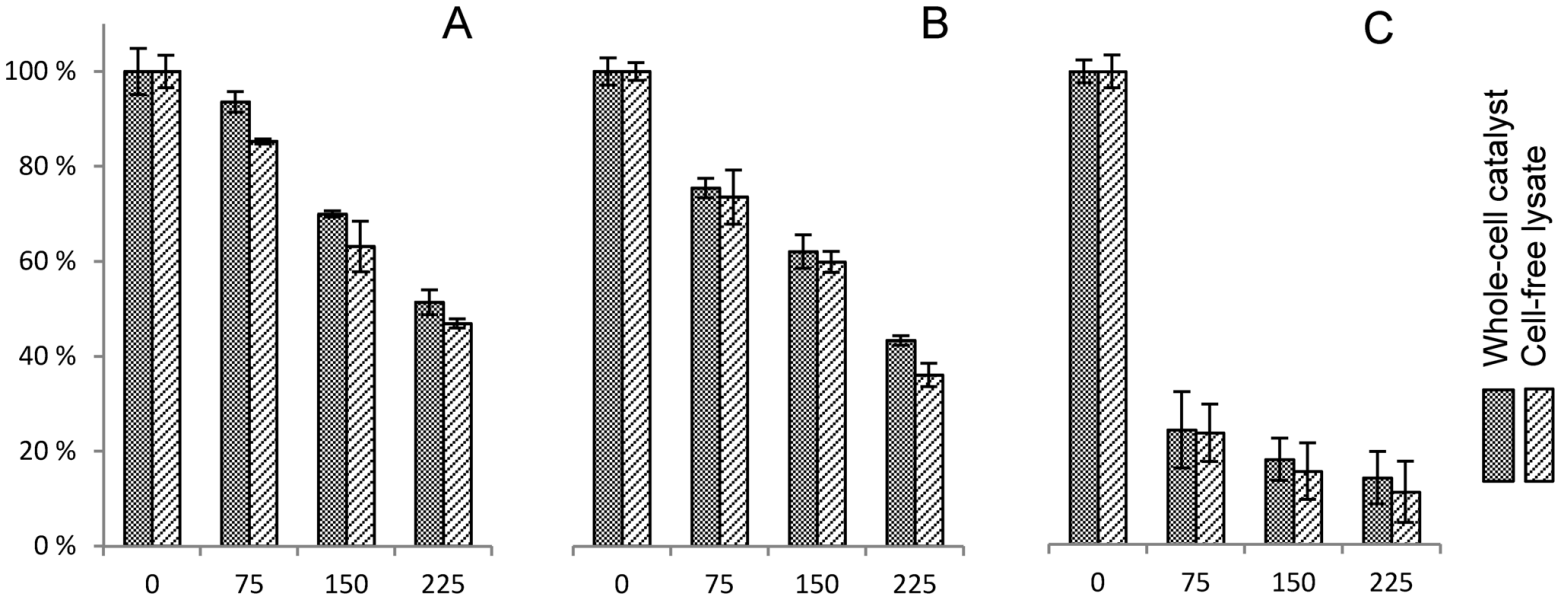
Inactivation of DERA whole-cell catalyst and DERA cell-free lysate with various aldehydes. Samples were treated with 75 mM, 150 mM and 225 mM substrate aldehydes for 15 minutes prior to the activity assay. The specific DERA activity was 226.8 kRFU s^−1^ g^−1^ and 226.6 kRFU s^−1^ g^−1^ for the whole-cell catalyst and for the cell-free lysate, respectively. Residual activities are given relative to non-treated whole-cell catalyst. Aldehydes used were acetaldehyde (A), **2b** (B) and **2g** (C).

Aldehyde-caused inactivation of DERA is a major problem in use of this enzyme for chemoenzymatic processes since it makes the reuse of the catalyst impossible [38], [61]–[62] and demands high loads for highly productive reactions [38], [44], [61]–[62]. Therefore, we were curious to see how the DERA whole-cell catalyst handles acetaldehyde **1** inactivation compared to the cell-free extract. In general, one can expect higher stability of proteins in their natural intercellular environment compared to cell-free extracts, due to the mechanisms organisms have, for dealing with missfolding issues [89], aggregation of proteins [90], oxidative stress [91], temperature stress [92], etc. We compared the inactivation rate of diluted DERA whole-cell catalyst and cell-free extract in presence of 75, 150 and 225 mM acetaldehyde **1** by measuring residual activity with the fluorometric method. A small, but consistent stabilization of enzyme activity is apparent from the results (Figure 4), indicating that the cellular environment may indeed have a beneficial effect on DERA stability.

### Whole-cell Enzymatic Synthesis of Lactonized Statin Side-chain Precursors

Batch processes (the whole amount of the substrates was added to the reaction at t = 0), using DERA whole-cell catalyst and 400 mmol L^−1^ of various, readily accessible, 2-substituted acetaldehydes (**2b, 2e, 2f** and **2g**) [83], [93]–[95] with 2.1 molar equivalents of acetaldehyde **1** were performed as described in the methods section. The GC-MS analysis confirmed accumulation of the expected corresponding lactols (**3b, 3e, 3f** and **3g**). The compounds were isolated and characterized with ^1^H-NMR, ^13^C-NMR and HRMS (Information S2). The confirmation of the structure of **3f** and **3g,** previously shown to be excellent starting material for super-statin synthesis [15]–[16], [78]–[81], [83], broadens the scope of molecules which can be obtained by DERA-catalyzed reactions, and this is the first time these molecules have been synthesized enzymatically. The successful production of the dimethoxy-substituted lactol **3f** for which unsuccessful synthesis attempts (using DERA) have been reported [57], was especially surprising. In agreement with Gijsen *et al*. [42], [57], significant amounts of 2,4,6-trideoxyhexose (**3a**) have been detected when only acetaldehyde **1** was used as a substrate for the reaction. Surprisingly, **3a** was also found accumulating as a rule (albeit in lesser quantities) in all other reactions, where apart from acetaldehyde **1**, 2-substituted acetaldehydes were used (Table 1). This was not emphasized to date, despite the numerous reports on the DERA-catalyzed sequential reactions using acetaldehyde **1** as one of the substrates [37]–[44], [46], [57], [59]–[60]. Although there is little value in this compound in terms of starting material for super-statin synthesis (due to lack of appropriate leaving group at the 6′ position, which would allow further transformations toward super-statin assembly), the finding is important. Namely, the chemical and physical properties of **3a** make the removal of this compound from the target cyclic hemiacetal side-chain products, obtained by DERA chemoenzymatic process, difficult during the work-up of the reactions. In addition, the accumulation rate of **3a** is an important indicator of the substrate availability during the reactions and was taken into account for optimization of substrate feeding.

**Table 1:** Reaction species in batch reactions using whole-cell catalyst and various substrate aldehydes.

Substrate (mmol L^−1^)	1	2	8	3a	3	10	*	Yield
**1** (840)	2.7 (19)	n.a	0.	136.2 (173)	**0**	0.6 (1)		62%
**2b** (400)+**1** (840)	7.5 (32)	0	4.3 (9)	9.5 (15)	**223.6 (258)**	5 (9)	22.5	65%
**2b** (400)+**1** (400)	0	11.2 (78)	27.0 (56)	0	**93.2 (108)**	2.8 (5)	40.6	27%
**2e** (400)+**1** (840)	0	150 (236)	80	91 (116)	**85 (43)**	3 (2)		11%
**2f** (400)+**1** (840)	0	88.6 (238)	52.2	85.7 (109)	**74.5 (54)**	2.4 (2)		14%
**2g** (400)+**1** (840)	0	50.0 (103)	76.9 (76)	32.6 (41)	**275.3 (182)**	13.7 (9)		45%
**2g** (400)+**1** (400)	0	77.8 (190)	98.2 (83)	2 (3)	**103.5 (80)**	4.7 (2)		20%

Whole-cell DERA catalyst was used (DERA specific activity = 232 kRFU s^−1^ g^−1^, WCW = 207 g L^−1^). Data are given as GC-FID peak area after 60 minutes after addition of the substrates, and calculated to molar concentration (mmol L^−1^, in brackets) where isolated reference material was available. There was no considerable change in reaction-species concentration after 60 min (reaction was followed up to 180 min) indicating loss of DERA activity early in the reaction.

* presumably 2,6-chloro-2,4-dideoxyhexose. GC-MS data for this compound are available in Information S8.

**Figure 5:**
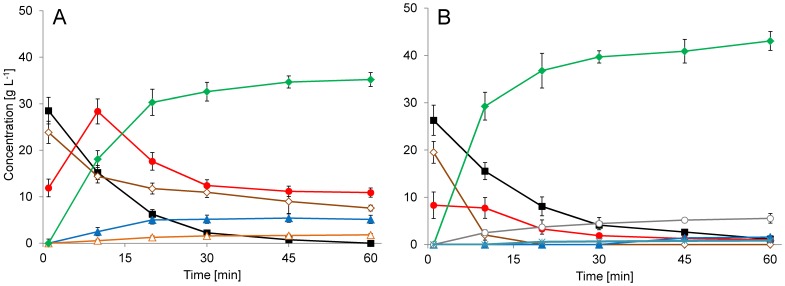
Time course of whole-cell, DERA-catalyzed batch reactions. Reactions were performed using *E. coli* BL21 (DE3) pET30/*deoC* fermentation cultures directly (DERA specific activity = 232 kRFU s^−1^ g^−1^, WCW = 207 g L^−1^). Results are given as mass concentrations obtained from GC-FID analysis. The measured quantity of a particular compound, with the exception of the stable 6-ring hemiacetals (**3**), represents the sum of the corresponding equilibrium forms (hydrate, aldehyde and acetal/hemiacetal), which exist under the reaction conditions. **A:** Reaction species data from reactions using 400 mmol L^−1^ of **2g** and 840 mmol L^−1^ of **1** are shown. **1** (▪, black), **3a** (▴, blue) **3g** (♦, green), **8g** (•, red), **10g** (Δ, orange) and **2g** (◊, brown). **B:** Reaction species data from reactions using 400 mmol L^−1^ of **2b** and 840 mmol L^−1^ of **1** are shown. **1** (▪, black), **3a** (▴, blue) **3b** (♦, green), **8b** (•, red), **10b** (Δ, orange), **2b** (◊, brown), 2,6-chloro-2,4-dideoxyhexose (□, grey). Concentration of the latter (Information S8) is evaluated based on the assumption, that the GC-FID response factor is similar to that of **3b**.

The yields of the described batch reactions in Table 1 are given as non-isolated yields (calculated from the GC-FID analysis of the reaction samples and purified, NMR-evaluated compound samples). The mass balance of the reactions, taking into account the unreacted substrates, was found to be short of the theoretical outcome. This led us to search for the missing substrate balance in either accumulating intermediates or side products. Since both whole broth and cleared lysates are enzymatically complex systems, a side reaction of some kind would not be surprising. The GC-MS analysis showed accumulation of compounds with the mass spectra in agreement with products of single aldol condensation (**8b, 8e, 8f** and **8g**) in the reaction mixture at considerable level (Figure 5a and 5b: the reactions with **2b** and **2g** are shown for illustration). The calculated yields of the reactions with the **2e** and the **2f** were well below those observed with **2b** and **2g** and no further attention was given to the former two. After purification and characterization (^1^H-NMR, ^13^C-NMR and HRMS) of **8g** (Information S3), the mass balance of the reaction with acetyloxy-acetaldehyde (**2g**) was re-assessed. More than 90% of the substrate **2g** was accounted for, either in the final product (**3g, 10g**) or in the intermediate (**8g**). Although accumulation of **8b** in certain reaction conditions was indicated before [42], the significant accumulation of **8** during all of the reactions was a surprising observation since no attention to this intermediate is given in the past optimizations of DERA processes [42]–[46], [59]–[60]. The observation offered another indicator by which the reaction conditions could be optimized.

Interestingly, an additional product of DERA was found accumulating in the reactions using **2b** as one of the substrates. The mass spectra of this compound indicate a product of condensation of two 2-chloro-acetyldehyde molecules with one acetaldehyde (Information S8). This new compound, presumably (4*R*,6*S*)-3-chloro-6-(chloromethyl)tetrahydro-2*H*-pyran-2,4-diol, was found accumulating in higher amounts in the reaction where 1 to 1 ratio between acetaldehyde and **2b** was used (Table 1).

Incidentally, a small amount of acetic acid was detected accumulating during the reactions with **2g** and acetaldehyde **1** in a rate not found in any of the reactions using **2b** as a substrate (Information S10). This finding points toward likely hydrolysis of **2g** to acetic acid and 2-hydroxy-acetaldehyde by a hydrolase present in the whole-cell catalyst. Indeed, incubation of **2g** alone with the whole-cell catalyst results in partial degradation and accumulation of acetic acid. Interestingly, we have also detected accumulation of trace quantities of a side product with mass spectra matching 2,4-dideoxy erythrose (data not shown). This molecule could originate from DERA-catalyzed condensation of acetaldehyde **1** and hydroxyl-acetaldehyde [42] arising from hydrolysis of **2g**. The above, were the only indications of a side activity in the whole-cell catalyst we have observed during our work.

An important finding, indicating transfer or diffusion of the reaction substrates, intermediates and products across the cellular envelopes, was made in the observation of practically identical reaction dynamics and yields when using either whole-cell catalyst or cell-free lysate with matched DERA activity for production of **3g** (data not shown). Since large majority of the DERA was found in the cellular fraction during the whole-cell process (Information S6), the reaction must proceed predominately inside the cells in order to match the reaction species dynamics observed with the cell-free extract process. Concentrations of the reaction substrates, intermediates and products in the acetonitrile-quenched reaction supernatant were found equal to intracellular concentrations obtained by sonication of the acetonitrile-quenched biomass pellet (GC-FID analysis). It is worth noting that the tightly packed biomass from the reaction mixtures represents ∼ 20% of the reaction volume and that error in the above observation due to presence of compounds in the intercellular space is unlikely. The temporal distribution of intracellular vs. extracellular concentrations shows no apparent effects in the reaction kinetics that could arise from diffusion limitations. The accumulation of the intermediate **8g** both intra- and extra-cellularly in the first part of the process and its later consumption, as well as the observations with the activity assay experiments, further support the finding.

### The Dynamics of the Reactions

**Figure 6:**
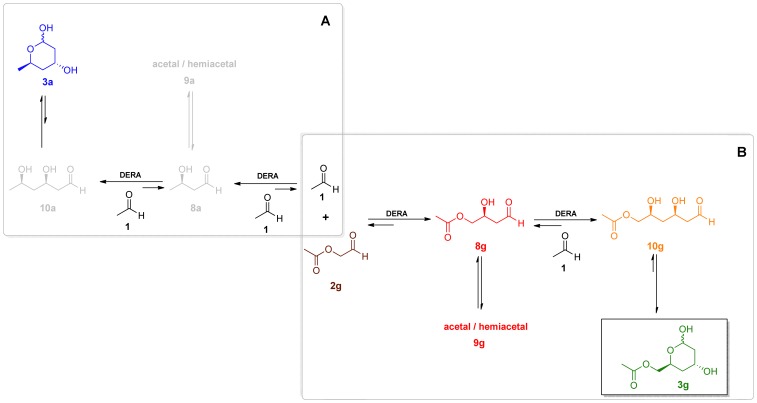
Compounds influencing the production of acetiloxy-lactol (3g). Hydrate forms of the aldehydes (**11, 12 and 13**) are not depicted here. **A:** Reaction species arising from acetaldehyde alone. **B:** Reaction species arising from acetaldehyde **1** and **2g.** Several acetal/hemiacetal (**9g**) species were found in equilibrium with **8g**. These may include monomeric cyclic hemiacetal as well as dimeric and trimeric cyclic acetals/hemiacetals (Information S3).

Due to the complex nature of the reaction wherein the activity of the catalyst is changing over time in a non-constant manner (inactivation of DERA) [38], [61]–[62], [96], occurring reactions are reversible (retro-aldol reaction) [54]–[55], the aldehyde substrates [53], intermediates and products are in equilibrium with their presumably nonreactive forms [42], [97]–[101], (Information S3 and S4), and one of the substrates is involved in a parallel reaction (formation of **3a**), in-depth understanding of the reaction dynamics is extremely difficult. Even more so, the complexity increases when considering existence of alternative double condensation products (Information S8) found in batch reactions with **2b**. A simplified scheme of the reaction species for a process with **2g** is illustrated in Figure 6.

The observed accumulation of the single-aldol-condensation intermediates **8** in the reactions can generally be explained by second-order kinetics for consecutive reactions [102]. The difference in the initial substrate concentrations for the first and second condensation alone can explain the effect, given similar kinetic constants for both steps. The accumulation rate of the intermediate **8**, however, was found to be significantly higher in reactions with **2g** compared to the ones using **2b** (Figure 5). Also, in comparison to the process with **2b**, the reaction yielding **3a** seems more competitive to the condensation converting **8g** to **3g**, in reactions using **2g**. In addition, high accumulation rates of the intermediate **8g** were observed when the substrates were fed continuously at a rate which results in concentration of the substrate **2g** near zero throughout the reaction (Information S9). The availability of **2g** for the enzyme is even lower since it was found predominantly in hydrate form in water (Information S4). Therefore, the rate of the second condensation must be lower, to allow the observed accumulation of the intermediate (**8g**). This can be attributed to markedly different kinetic constant values for the first and the second condensation step due to DERA properties, or to the equilibriums between the aldehyde, acetal/hemiacetal and hydrate forms of the reaction species (Figure 2).

In fact the equilibrium between the reaction product **3** and its open, aldehyde form (**10**), is considered to be the reason why the sequential aldol reaction stops at the second condensation (the equilibrium is strongly shifted toward **3**) [42], [57]. Surely, one possibility that could influence the rate of the second condensation, is the role of acetal or hemiacetal (**9**) equilibrium forms originating from the single-condensation intermediate (**8**) under the reaction conditions.

Indeed, although the reaction species (with exception of stable 6-ring hemiacetals **3**) are observed only in the aldehyde form in the GC analysis, the ^1^H-NMR and ^13^C-NMR spectra for purified **8g** showed (in addition to the expected hydrate form **12g**) presence of several distinct acetal-specific signals under mild conditions. Due to the number of the acetal signals observed for these equilibrium forms, which we collectively termed **9**, indisputable evidence on their structures remains elusive. According to the published examples, the species **9** may include cyclic lactol [42], dimeric lactol [97], dimeric hemiacetal [98] or cyclic trioxane [99] (Information S3).

Shifts in equilibrium between **8g**, **9g** and **12g** can be observed when various solvents are used (D_2_O, CDCl_3_, DMSO-d_6_) for the ^1^H-NMR and ^13^C-NMR measurements. The equilibrium is shifted toward **8g** and **9g** in nonpolar CDCl_3_ whereas the hydrate **12g** is predominant in polar solvents, although aldehyde **8g** and acetal-specific proton signals of **9** can also be observed (Information S3). In contrast, only hydrate and aldehyde species are found for the initial substrates **1** and **2g** in D_2_O as indicated by ^1^H-NMR (Information S4).

Therefore, in analogy with DERA-catalyzed coupling of hydroxyacetaldehyde and acetaldehyde **1**, where the formation of stable hemiacetals both with the initial substrate and the product of the first aldol condensation was found preventing successive reaction steps [42], one can foresee a similar, yet less prominent effect influencing the second condensation to form **3g.**

On the other hand, we can predict but one difference between **8b** and **8g** in the possibility of formation of cyclic acetals/hemiacetals. The presence of the acetyl group in **8g** changes the possible diversity in these equilibria compared to the situation with **8b** (Information S3). Although the reversible rearrangement (transesterification) of **8g**, leading to a five membered lactol, could reduce the availability of the mono-aldol intermediate to the enzyme, we cannot claim that this is the sole reason for the observed lower second condensation rate compared to the reactions with the chloro-substituded counterparts. Still, consideration of the existence of the equilibrium forms of the intermediates offers an insight, complementary to the mechanistic studies published for DERA [52]–[53], [61], when interpreting the dynamics of sequential aldol condensations.

Next, analysis of the residual DERA activity, determined for washed whole-cell samples taken from the batch reaction with 400 mmol L^−1^ **2g** and 840 mmol L^−1^ acetaldehyde **1**, showed rapid inactivation of DERA. Only 28% of the initial activity was detected after the first 10 minutes of the reaction, 18% after 30 minutes and less than 15% after 1 h (Information S7). Reliable residual activity measurements for the parallel cell-free extract reaction could not be performed due to the lack of a convenient method for removal of the inactivating aldehydes present in the assay samples. This rapid loss of enzyme activity was another observation calling for optimization of the feeding strategy, although it was not a surprising find. The issue has already been addressed by Greenberg *et* *al*. [44]. In their report, chloro-acetaldehyde (**2b**) was found to be significantly more inhibiting compound compared to acetaldehyde **1**. In our hands, derived from the results where the residual activity of the whole-cell catalyst in presence of various aldehydes was compared, acetaldehyde **1** was found to be almost as potent inactivator of DERA as **2b**. On the other hand, addition of **2g**, the prime focus of our interest, resulted in significantly higher inactivation rates making the development of a highly productive process with this substrate even more challenging (Figure 4). For this reason, and in light of the accumulating **8** and **3a** in the reaction, as well as the formation of alternative products such as the 2,6-chloro-2,4-dideoxyhexose (Figure 5, Information S8), it is obvious that the feeding strategy described previously (proposing addition of the two reactants in a constant 1∶2.1 molar ratio) [44], is not generally applicable to the DERA-catalyzed processes and can be improved by controlling feeding rates of each individual substrate throughout the reaction.

### Fed-batch Whole-cell Process

Using a semi-empiric approach, various feeding profiles for each of the two substrates (**1** and **2g**) were tested in fed-batch reactions using DERA whole-cell catalyst (data not shown). When using a constant molar ratio (**2g** : **1** = 1 : 2.1) of the substrates, similarly to the batch process described above (Figure 5a), the intermediate **8g** was formed much faster than was consumed to form **3g**. Consequently, a surplus of acetaldehyde was created, resulting in enhanced accumulation of **3a**. At the end, rapid loss of DERA activity and lack of acetaldehyde (consumed for **3a** formation) lead to unconverted **8g** and low yields. As the next step, with the aim to control the accumulation of **3a**, we set up fed-batch reactions with addition of the total quantity of **2g** at the start of the reaction. Acetaldehyde was fed in a profile that allowed consumption of the accumulated **8g** almost completely. Although this feeding strategy showed high yields, and both **3a** and **8g** levels below 5% each, the inactivation of DERA prevented high yields in processes with **2g** loads higher than 400 mmol L^−1^.

**Figure 7:**
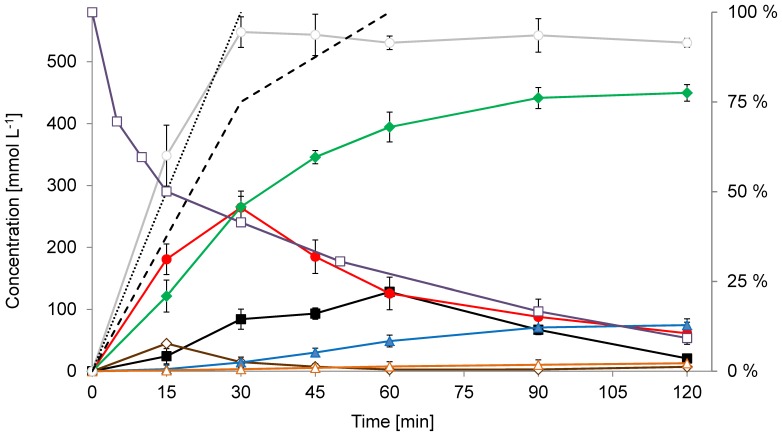
Time course of whole-cell, DERA-catalyzed, fed-batch reactions yielding 3g. Reaction species data from three independent experiments using (in total) 550 mmol L^−1^ of **2g** and 1200 mmol L^−1^ of **1** are shown. Whole-cell catalyst (*E. coli* BL21 (DE3) pET30/*deoC* high-density culture) with 217 kRFU s^−1^ g^−1^ DERA specific activity and 182 g L^−1^ WCW was used. Results are given as molar concentrations obtained from GC-FID analysis. The measured quantity of a particular compound, with the exception of the stable 6-ring hemiacetals (**3**), represents the sum of the corresponding equilibrium forms (hydrate, aldehyde and acetal/hemiacetal) which exist under the reaction conditions. **1** (**▪**, black), **3a** (▴, blue) **3g** (♦, green), **8g** (•, red), **10g** (Δ, orange), **2g** (◊, brown), cumulative molarity of reaction species originating from **2g** (□, grey; sum of **2g**, **8g**, **10g** and **3g** ). Secondary vertical axis shows in %: residual DERA activity (□, violet), cumulative feed of **2g** (dotted line), cumulative feed of **1** (dashed line).

Finally, a compromise was made in the feeding strategy (Figure 7), taking into account the indications to the reaction dynamics we have obtained. The ratio between **2g** and acetaldehyde was adjusted to 1 : 2.2 in order to compensate for the acetaldehyde consumed in **3a** formation. **2g** was fed faster than acetaldehyde **1** and only in the first 30 minutes of the process. This resulted in a higher rate of **3g** formation in the first part of the process compared to the strategy where all of the **2g** was added at the beginning. At the same time, the formation of **3a** was low. As **2g** was being consumed, a surplus of acetaldehyde was created, driving both conversion of **8g** to **3g** and formation of **3a**. The remaining 25% of the acetaldehyde (fed in the next 30 minutes) allowed efficient conversion of the large part of the mono-aldol intermediate in a reasonable timeframe of the remaining 60 minutes, although some **3a** was formed due to this acetaldehyde surplus.

**Figure 8:**
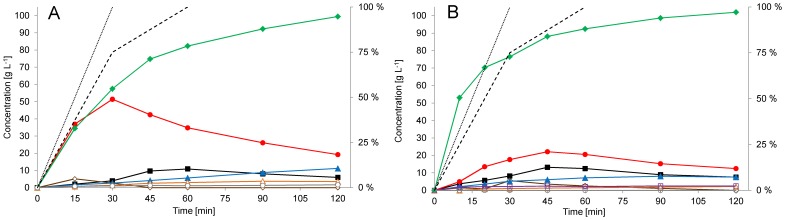
Time course of exemplary whole-cell, DERA-catalyzed, fed-batch reactions with ∼50 g L^−1^ h^−1^ volumetric productivity. Whole-cell catalyst (E. coli BL21 (DE3) pET30/deoC high-density culture) with 247 kRFU s^−1^ g^−1^ DERA specific activity and 215 g L^−1^ WCW was used. Results are given as mass concentrations obtained from GC-FID analysis. The measured quantity of a particular compound, with the exception of the stable 6-ring hemiacetals (**3**), represents the sum of the corresponding equilibrium forms (hydrate, aldehyde and acetal/hemiacetal) which exist under the reaction conditions. **A:** Reaction species data from reaction using (in total) 700 mmol L^−1^ of **2g** and 1540 mmol L^−1^ of **1** are shown. **1** (▪, black), **3a** (▴, blue) **3g** (♦, green), **8g** (•, red), **10g** (Δ, orange), **2g** (◊, brown), acetic acid (□, grey). Secondary vertical axis shows in %: cumulative feed of **2g** (dotted line), cumulative feed of **1** (dashed line). **B:** Reaction species data from reaction using (in total) 700 mmol L^−1^ of **2b** and 1540 mmol L^−1^ of **1** are shown. **1** (▪, black), **3a** (▴, blue) **3b** (♦, green), **8b** (•, red), **10b** (Δ, orange), **2b** (◊, brown), acetic acid (□, grey), 2,6-chloro-2,4-dideoxyhexose (□, purple). Secondary vertical axis shows in %: cumulative feed of **2b** (dotted line), cumulative feed of **1** (dashed line).

More than 20 fed batch reactions with **2g** loads between 500 mmol L^−1^ and 700 mmol L^−1^ were made using this feeding profile and titers of 100 g L^−1^ of **3g** were reached (103.2 g L^−1^ including the open form **10g**) in 2 h reaction time using 700 mmol L^−1^ of **2g** (Figure 8). Due to higher process robustness and higher yields (78–86%), we chose to use lower substrate loads (550 mmol L^−1^ of **2g**), which routinely resulted in 85–91 g L^−1^ titers of **3g** in 120 min (Figure 7). The chromatographic purity of the reaction-mixture samples at harvest time were 82%–86% and crude **3g** prepared with whole-broth extraction from these reactions had >70% assay for the **3g** as a rule. The volumetric productivity of the process was over 40 g L^−1 ^h^−1^. Despite the optimization, accumulation of **3a** and unreacted intermediate (**8g**) could not be completely avoided. **3a** and **8g** were found, in average, in 10% (w/w) each, compared to **3g**. The ratio between **3g** and its open, aldehyde form (**10g**) was constant at the reaction conditions (∼ 3% w/w) but could be influenced in favor of **10g** by lowering the pH. Residual activity of DERA during the reaction was tested and although <10% of the activity was detected at the completion of the reaction, the inactivation profile shows significant improvement compared to the batch process (Information S7). Using the fed-batch process ∼ 45% of the activity remained after 30 minutes and ∼ 25% after 60 min of the process even with 550 mmol L^−1^ load of **2g** (Figure 7).

For validation of applicability of the process for preparation of other substituted lactols, chlorolactol (**3b**) reaction was selected due to the challenging precedents in the literature [44], [60]. Using the same conditions as used for production of **3g**, with 700 mmol L^−1^ of **2b**, 102 g L^−1^ of **3b** (104.2 g L^−1^ including the open form **10b**) was prepared in 120 min (88% yield, volumetric productivity 51 g L^−1^h^−1^ ). Reaction species profiles were found to be similar to the acetyloxylactol (**3g**) process, however - as expected, the accumulation of the mono-aldol intermediate **8b** was less pronounced in the reaction with **2b** resulting in higher production rates in the first part of the process (Figure 8). Compared to the batch process, formation of 2,6-chloro-2,4-dideoxyhexose was at barely detectable levels.

The resulting process not only improves the volumetric productivity reported for the w.t. *E. coli* DERA catalyzed production of **3b** [42], [44], [60], but also achieves this with a considerably cheaper biocatalyst. The material cost contribution of the whole-cell catalyst is estimated to be in the range of 2.0–3.5 € per assay kilogram of the crude isolate of chiral lactols (depending on the target titer). Furthermore, the fermentation and the chemoenzymatic process were joined into a one-pot telescope process, additionally simplifying the industrialization.

### Preparation of Advanced Super-statin Intermediates

The conversion of the isolated lactols **3** to a useful statin side chain can be achieved by oxidation of lactols **3** to the lactones **15,** followed by silyl protection of 4-hydroxyl substituent resulting in compound **5** [78], an advanced intermediate, recently described *en route* to preparation of rosuvastatin [15], [80], [81] and pitavastatin [16], [80], [81]. The lactol oxidation using various oxidation methods such as Br_2_/BaCO_3_ [103], *N*-iodosuccinimide [104], Ag_2_CO_3_ [105], Pt/C-O_2_ [106], RuCl_2_(PPh_3_)_3_/cyclohexanone [107], MnO_2_ [108], or NaOCl/AcOH [60] was described before. Aiming toward a scalable and sustainable process we tested several process options in order to use the whole-cell DERA reaction mixtures in a straightforward manner (Table 2). Initially, oxidation of **3g** using bromine in the presence of barium carbonate was used to obtain the corresponding lactone **4** in good 78% yield (Table 2, entry 1). Prior to oxidation, addition of acetonitrile to the whole-cell reaction mixture and filtration of the precipitate was used to remove majority of the cells and cell debris. Inconveniently, the remaining proteins, carbohydrates and other organic material from the extract, as well as the presence of undesired compounds **3a** and **8g**, make the oxidation reaction more difficult as it requires 3.3 equivalents of bromine to fully oxidize the lactol **3g**. Even more equivalents were needed when chemoenzymatic reaction mixtures having higher amounts of **3a** and **8g** were used. To avoid the large amount of hazardous and toxic bromine needed, the process using bleach [60] was tested and modified (avoiding the solvent exchange) with the use of a mixture of aqueous NaH_2_PO_4_ solution and ethylacetate instead of acetic acid as solvent. This reaction also required large excess of the oxidant to reach completion of the reaction (6.5 eq.) and led to a similar, 77% yield (Table 2, entry 2).

**Table 2:** The optimization of lactol (**3g**) oxidation yielding lactone **15**.

	Cell removalmethod	Oxidant (eq.)	Additive	Yield
1	MeCN precipitation	Br_2_ (3.3)	BaCO_3_	78%
2	MeCN precipitation	NaOCl (6.5)	NaCl/NaH_2_PO_4_	77%
3	None	NaOCl (6.5)	NaCl/NaH_2_PO_4_	84%
4	Centrifugation	NaOCl (6.5)	NaCl/NaH_2_PO_4_	84%
5	Centrifugation	Ca(OCl)_2_ (9.5)	NaCl/NaH_2_PO_4_	64%
6	Centrifugation	Ca(OCl)_2_ (4.7)	NaCl/H_3_PO_4_	78%
7	None	Ca(OCl)_2_ (2.5)	NaCl/H_3_PO_4_	84%

In order to avoid the use of large amounts of acetonitrile for the removal of the biomass, the oxidation was tested directly on the whole-cell catalyst reaction mixture and on the centrifuged reaction mixture. Both reactions gave comparable and better, 84% yields (Table 2, entries 3 and 4) when the acetonitrile was omitted. An issue with the bleach oxidation process was raised upon scale-up; the large volume of water was introduced to the reaction due to the low bleach concentration. To address this issue, we decided to replace bleach with its solid equivalent calcium hypochlorite. At first, the reaction required larger amounts of the oxidant (Table 2, entry 5) and gave lower yield mostly because of filtration problems during the work-up. The replacement of sodium phosphate with phosphoric acid, while maintaining the pH at 3, led to reduction of oxidant usage and made the work-up filtration easier. This resulted in higher yield (Table 2, entry 6) and surprisingly improvements in both the amount of oxidant used and yield (84%) were achieved when using the whole-cell catalyst reaction mixture directly (entry 7). The lactone **15g** (oil) was isolated from the ethyl-acetate phase and purification of impurities derived mainly from compound **3a** was not possible except by chromatography. None the less, the lactone **15g** was used without purification and protected in standard conditions with TBDMSCl in dichloromethane in presence of imidazole to give **5** and then further converted to the compound **6** using previously reported procedure [80], , to obtain crystalline material in an overall yield of 62% for the three steps. The crystallization of **6** allowed removal of the impurities originating from the whole-cell chemoenzymatic process and carried over through oxidation and silylation steps. ^1^H-NMR and GC analysis of **6** showed very high purity (99.8% GC purity). Enantiomeric and diastereoisomeric purity was determined to be similar as previously reported (>99.9% *ee*, >99.8% *de*, Information S5) [80], [84]. Combined, the results show an efficient way of converting the lactols **3** derived from the whole-cell DERA catalyzed reactions in further steps toward statins. To this end, we avoided problematic and technically demanding downstream procedures yielding an especially low cost, scalable and green process with good overall yields. Again, the importance of controlling the DERA reaction intermediates and side products such as **8g** and **3a** (respectively) can be stressed out; this time not from the reaction yield point of view, but rather in terms of influence to the following chemical steps, namely the oxidation, silylation and deacetylation. The lactol **3a**, for example, is not problematic *per se* since it is efficiently removed in the late steps. The additional amount of TBDMSCl needed at the silylation step due to its presence, however, brings unnecessary extra costs to the process.

## Conclusions

The described process for production of chiral, lactonized super-statin intermediates, using a whole-cell DERA catalyst derived directly from a fed-batch, high-density fermentation with *E. coli* BL21 (DE3) overexpressing native *E. coli deoC,* shows unprecedented productivity and is highly cost effective. Volumetric productivities of 50 g L^−1^ h^−1^, with >80% yield and >80% chromatographic purity have been achieved with this process for 6′-chloro and 6′-acetyloxy substituted lactols. Moreover, ((2*S*,4*R*)-4,6-dihydroxytetrahydro-2*H*-pyran-2-yl)methyl acetate and (4*R*,6*S*)-6-(dimethoxymethyl)tetrahydro-2*H*-pyran-2,4-diol have been prepared, using DERA catalysis, for the first time. Although new insights into the dynamics of the reaction intermediates and side products are presented in this work, the complexity of the reaction leaves room for additional improvement of understanding of the DERA-catalyzed sequential aldol condensation reactions. This is especially true for the role of equilibria of the reaction aldehydes with their hydrate form on the one side and acetal/hemiacetal forms on the other. In addition, the dynamics of the availability of the substrates, defined by the feeding strategy and equilibria with their non-reactive forms in combination with substrate preference of the DERA enzyme, can lead the reaction to form completely unexpected products such as 2,6-chloro-2,4-dideoxyhexose. Nevertheless, excellent enantiomeric purities (*ee* >99.9%) which were demonstrated on downstream advanced intermediates allow the products obtained by the described process to fit directly into our recently described lactone pathway to optically pure super-statins. The enzymatic synthesis of ((2*S*,4*R*)-4,6-dihydroxytetrahydro-2*H*-pyran-2-yl)methyl acetate is the last piece in design of an efficient and industrially scalable synthesis of super-statins based on the direct coupling of lactonised diol side-chains to the heterocyclic part of the molecule.

## Supporting Information

Information S1 **Fermentation data.**
(PDF)

Information S2 **NMR, HR-MS, GC-FID and GC-MS data.**
(PDF)

Information S3 **NMR, HR-MS, GC-FID and GC-MS identification of the mono-aldol intermediates (8, 9, 12).**
(PDF)

Information S4 **Proton NMR of aldehyde precursors 1 and 2g in D_2_O.**
(PDF)

Information S5 **Determination of stereochemical purity.**
(PDF)

Information S6 **Comparison of DERA activity in the whole broth, washed cells and cell-free lysate with the assay mixture supernatant. DERA assay on whole cells.**
(PDF)

Information S7 **Residual DERA activity measurements and DERA distribution in the whole cell process with 2g.**
(PDF)

Information S8 **Proposed alternative double condensation product observed in reactions with 2b.**
(PDF)

Information S9 **A fed-batch process keeping 2g concentration near dynamic zero.**
(PDF)

Information S10 **Accumulation of acetic acid in reactions with 2g.**
(PDF)

## References

[1] Tobert JA (2003) Lovastatin and beyond: the history of the HMG-COA reductase inhibitors. Nat Rev Drug Discov 2: 517–526. 12815379 10.1038/nrd1112

[2] Istvan ES, Deisenhofer J (2001) Structural mechanism for statin inhibition of HMG-CoA reductase. Science 292: 1160–1164. 11349148 10.1126/science.1059344

[3] Brautbar A, Ballantyne CM (2011) Pharmacological strategies for lowering LDL cholesterol: statins and beyond. Nat Rev Cardiol 8: 253–265. 21321561 10.1038/nrcardio.2011.2

[4] Kidd J (2006) Life after statin patent expires. Nat Rev Drug Discov 5: 813–814. 17078172 10.1038/nrd2156

[5] Almuti K, Rimawi R, Spevack D, Ostfeld RJ (2006) Effects of statins beyond lipid lowering: potential for clinical benefits. Int J Cardiol 109: 7–15. 16054715 10.1016/j.ijcard.2005.05.056

[6] Switzer JA, Hess DC (2006) Statins in stroke: prevention, protection and recovery. Expert Rev Neurotherapeutics 6: 192–202. 10.1586/14737175.6.2.195 16466299

[7] Lahera V, Goicoechea M, de Vinuesa SG, Miana M, de las Heras N, et al (2007) Endothelial dysfunction, oxidative stress and inflammation in atherosclerosis: beneficial effects of statins. Curr Med Chem 14: 243–248. 17266583 10.2174/092986707779313381

[8] Paraskevas KI, Tzovaras AA, Briana DD, Mikhailidis DP (2007) Emerging indications for statins: a pluripotent family of agents with several potential applications. Curr Pharm Des 13: 3622–3636. 18220799 10.2174/138161207782794194

[9] Bersano A, Ballabio E, Lanfranconi S, Mazzucco S, Candelise L, et al.. (2008) Statins and stroke. Curr Med Chem 15: 2380–2392 and references cited therein. 10.2174/092986708785909139 18855667

[10] Endo A, Kuroda M, Tsujita Y (1976) ML-236A, ML-236B, and ML-236C, new inhibitors of cholesterogenesis produced by *Penicillium citrinum.* . J Antibiot 29: 1346–1348. 1010803 10.7164/antibiotics.29.1346

[11] Endo A (1992) The discovery and development of HMG-CoA reductase inhibitors. J Lipid Res 33: 1569–1582. 1464741

[12] Endo A, Hasumi K (1993) HMG-CoA reductase inhibitors. Nat Prod Rep 10: 541–550. 8121646 10.1039/np9931000541

[13] Endo A (2008) A gift from nature: the birth of the statins. Nature Med 14: 1050–1052. 18841147 10.1038/nm1008-1050

[14] Časar Z (2010) Historic overview and recent advances in the synthesis of super-statins. Curr Org Chem 14: 816–845.

[15] Časar Z, Steinbücher M, Košmrlj J (2010) Lactone pathway to statins utilizing the Wittig reaction. The synthesis of rosuvastatin. J Org Chem 75: 6681–6684. 20815370 10.1021/jo101050z

[16] Fabris J, Časar Z, Gazić Smilović I (2012) The use of a lactonized statin side-chain precursor in a concise and efficient assembly of pitavastatin. Synthesis 44: 1700–1710.

[17] Vasić-Rački ? (2006) History of industrial biotransformations - Dreams and realities. In: Liese A, Seelbach K, Wandrey C, editors. Industrial Biotransformations 2nd ed. Weinheim: Wiley-VCH. 1–29.

[18] Liese A, Villela Filho M (1999) Production of fine chemicals using biocatalysis. Curr Opin Biotechnol 10: 595–603. 10600695 10.1016/s0958-1669(99)00040-3

[19] Schulze B, Wubbolts MG (1999) Biocatalysis for industrial production of fine chemicals. Curr Opin Biotechnol 10: 609–615. 10600696 10.1016/s0958-1669(99)00042-7

[20] Wandrey C, Liese A, Kihumbu D (2000) Industrial biocatalysis: past, present, and future. Org Process Res Dev 4: 286–290.

[21] Zaks A (2001) Industrial biocatalysis. Curr Opin Chem Biol 5: 130–136. 11282338 10.1016/s1367-5931(00)00181-2

[22] Koeller KM, Wong C-H (2001) Enzymes for chemical synthesis. Nature 409: 232–240. 11196651 10.1038/35051706

[23] Schmid A, Dordick JS, Hauer B, Kiener A, Wubbolts M, et al (2001) Industrial biocatalysis today and tomorrow. Nature 409: 258–268. 11196655 10.1038/35051736

[24] Straathof AJJ, Panke S, Schmid A (2002) The production of fine chemicals by biotransformations. Curr Opin Biotechnol 13: 548–556. 12482513 10.1016/s0958-1669(02)00360-9

[25] Patel RN (2002) Microbial/enzymatic synthesis of chiral intermediates for pharmaceuticals. Enzyme Microb Technol 31: 804–826.

[26] Bertau M (2002) Novel developments in biocatalytic organic chemistry. Curr Org Chem 6: 987–1014.

[27] Schoemaker HE, Mink D, Wubbolts MG (2003) Dispelling the myths-biocatalysis in industrial synthesis. Science 299: 1694–1697. 12637735 10.1126/science.1079237

[28] Yazbeck DR, Martinez CA, Hu S, Tao J (2004) Challenges in the development of an efficient enzymatic process in the pharmaceutical industry. Tetrahedron: Asymmetry 15: 2757–2763.

[29] Panke S, Held M, Wubbolts M (2004) Trends and innovations in industrial biocatalysis for the production of fine chemicals. Curr Opin Biotechnol 15: 272–279. 15357999 10.1016/j.copbio.2004.06.011

[30] Panke S, Wubbolts M (2005) Advances in biocatalytic synthesis of pharmaceutical intermediates. Curr Opin Chem Biol 9: 188–194. 15811804 10.1016/j.cbpa.2005.02.007

[31] Pollard DJ, Woodley JM (2006) Biocatalysis for pharmaceutical intermediates: the future is now. Trends Biotechnol 25: 66–73. 17184862 10.1016/j.tibtech.2006.12.005

[32] Hudlicky T, Reed JW (2009) Applications of biotransformations and biocatalysis to complexity generation in organic synthesis. Chem Soc Rev 38: 3117–3132. 19847346 10.1039/b901172m

[33] Müller M (2005) Chemoenzymatic Synthesis of Building Blocks for Statin Side Chains. Angew Chem Int Ed 44: 362–365. 10.1002/anie.200460852 15593081

[34] Liljeblad A, Kallinen A, Kanerva LT (2009) Biocatalysis in the Preparation of the Statin Side Chain. Curr Org Synth 6: 362–379.

[35] Ma SK, Gruber J, Davis C, Newman L, Gray D, et al (2010) A green-by-design biocatalytic process for atorvastatin intermediate. Green Chem 12: 81–86.

[36] Schürman M, Wolberg M, Panke S, Kierkels H (2010) The development of short, efficient, economic, and sustainable chemoenzymatic processes for statin side chains. In: Dunn PJ, Wells AS, Williams MT, editors. Green Chemistry in the Pharmaceutical Industry 1st ed. Weinheim: Wiley-VCH. 127–144.

[37] Whalen LJ, Wong C-H (2006) Enzymes in organic synthesis: aldolase-mediated synthesis of iminocyclitols and novel heterocycles. Aldrichim Acta 39: 63–71.

[38] Dean SM, Greenberg WA, Wong C-H (2007) Recent Advances in Aldolase-Catalyzed Asymmetric Synthesis. Adv Synth Catal 349: 1308–1320.

[39] Samland AK, Sprenger GA (2006) Microbial aldolases as C–C bonding enzymes-unknown treasures and new developments. Appl Microbiol Biotechnol 71: 253–264. 16614860 10.1007/s00253-006-0422-6

[40] Bolt A, Berry A, Nelson A (2008) Directed evolution of aldolases for exploitation in synthetic organic chemistry. Arch Biochem Biophys 474: 318–330. 18230325 10.1016/j.abb.2008.01.005 PMC2431125

[41] Clapés P, Fessner W-D, Sprenger GA, Samland AK (2010) Recent progress in stereoselective synthesis with aldolases. Curr Opin Chem Biol 14: 154–167. 20071212 10.1016/j.cbpa.2009.11.029

[42] Gijsen HJM, Wong C-H (1994) Unprecedented asymmetric aldol reactions with three aldehyde substrates catalyzed by 2-deoxyribose-5-phosphate aldolase. J Am Chem Soc 116: 8422–8423.

[43] Gijsen HJM, Qiao L, Fitz W, Wong C-H (1996) Recent advances in the chemoenzymatic synthesis of carbohydrates and carbohydrate mimetics. Chem Rev 96: 443–474. 11848760 10.1021/cr950031q

[44] Greenberg W, Varvak A, Hanson SR, Wong K, Huang H, et al (2004) Development of an efficient, scalable, aldolase-catalyzed process for enantioselective synthesis of statin intermediates. Proc Natl Acad Sci USA 101: 5788–5793. 15069189 10.1073/pnas.0307563101 PMC395986

[45] Ikunaka M (2007) Catalytic asymmetric carbon-carbon bond formations: their evolution from biocatalysis to organocatalysis over the millennium. Org Process Res Dev 11: 495–502.

[46] Yoshida Y, Noritake T, Watanabe M, Nakamoto Y (2005) Method for producing (4R,6S)-6-benzyloxymethyl-4-hydroxy-tetrahydro-2-pyrone. (Ube Industries, Ltd., Japan). Jap Pat Appl JP 2005229858 A Chem Abstr (2005) 143: 228047.

[47] Časar Z, Mesar M, Kopitar G, Mrak P, Ošlaj M (2008) Synthesis of statins. (Lek Pharmaceuticals d.d.). PCT Int Appl WO 2008119810 A2 Chem Abstr (2008) 149: 448104.

[48] Machajewski TD, Wong C-H (2000) The catalytic asymmetric aldol reaction. Angew Chem Int Ed 39: 1352–1374. 10.1002/(sici)1521-3773(20000417)39:8<1352::aid-anie1352>3.0.co;2-j 10777624

[49] Mestres R (2004) A green look at the aldol reaction. Green Chem 6: 583–603.

[50] Sukumaran J, Hanefeld U (2005) Enantioselective C-C bond synthesis catalysed by enzymes. Chem Soc Rev 34: 530–542. 16137165 10.1039/b412490a

[51] Mlynarski J, Paradowska J (2008) Catalytic asymmetric aldol reactions in aqueous media. Chem Soc Rev 37: 1502–1511. 18648676 10.1039/b710577k

[52] Heine A, DeSantis G, Luz JG, Mitchell M, Wong C-H, et al (2001) Observation of covalent intermediates in an enzyme mechanism at atomic resolution. Science 294: 369–374. 11598300 10.1126/science.1063601

[53] Heine A, Luz JG, Wong C-H, Wilson IA (2004) Analysis of the class I aldolase binding site architecture based on the crystal structure of 2-deoxyribose-5-phosphate aldolase at 0.99 A° resolution. J Mol Biol 343: 1019–1034. 15476818 10.1016/j.jmb.2004.08.066

[54] Racker E (1952) Enzymatic synthesis and breakdown of deoxyribose phosphate. J Biol. Chem. 196: 347–365. 12980976

[55] Pricer WE, Horecker BL (1960) Deoxyribose aldolase from Lactobacillus plantarum. J Biol Chem 235: 1292–1298. 14434864

[56] Barbas CF, Wang Y-F, Wong C-H (1990) Deoxyribose-5-phosphate aldolase as a synthetic catalyst. J Am Chem Soc 112: 2013–2014.

[57] Gijsen HJM, Wong C-H (1995) Sequential three- and four-substrate aldol reactions catalyzed by aldolases. J Am Chem Soc 117: 7585–7591.

[58] Chen L, Dumas PD, Wong CH (1992) Deoxyribose-5-phosphate aldolase as a catalyst in asymmetric aldol condensation. J Am Chem Soc 114: 741–748.

[59] Kierkels JGT, Mink D, Panke S, Lommen FAM, Heemskerk D (2003) Process for the preparation of 2,4-dideoxyhexoses and 2,4,6-trideoxyhexoses. (DSM N.V.). PCT Int Appl WO 2003006656 A2 Chem Abstr (2003) 138: 112523.

[60] Greenberg W, Wong K, Varvak A, Swanson R (2004) Chemoenzymatic methods for the synthesis of statins and statin intermediates. (Diversa Corporation). , PCT Int Appl WO 2004027075 A2 Chem Abstr (2004) 140: 302423.

[61] Jennewein S, Schürmann M, Wolberg M, Hilker I, Luiten R, et al (2006) Directed evolution of an industrial biocatalyst: 2-deoxy-D-ribose 5-phosphate aldolase. Biotechnol J 1: 537–548. 16892289 10.1002/biot.200600020

[62] Habeeb AFSA, Hiramoto R (1968) Reaction of proteins with glutaraldehyde. Arch Biochem Biophys 126: 16–26. 4174905 10.1016/0003-9861(68)90554-7

[63] Franke D, Hsu C-C, Wong C-H (2004) Directed evolution of aldolases. Methods Enzymol 388: 224–238. 15289075 10.1016/S0076-6879(04)88020-0

[64] Suau T, Calveras J, Clapés P, Benaiges MD, Álvaro G (2005) Immobilization of fuculose-1-phosphate aldolase from *E. coli* to glyoxal-agarose gels by multipoint covalent attachment. Biocatal Biotransfor 23: 241–250.

[65] Suau T, Álvaro G, Benaiges MD, López-Santín J (2009) Performance of an immobilized fuculose-1-phosphate aldolase for stereoselective synthesis. Biocatal Biotransfor 27: 136–142.

[66] Wang A, Wang M, Wang Q, Chen F, Zhang F, et al (2011) Stable and efficient immobilization technique of aldolase under consecutive microwave irradiation at low temperature. Bioresour Technol 102: 469–474. 20843684 10.1016/j.biortech.2010.08.048

[67] Faber K (2011) Biotransformations in Organic Chemistry: A Textbook 6th ed. Berlin: Springer: 9–10.

[68] Ishige T, Honda K, Shimizu S (2005) Whole organism biocatalysis. Curr Opin Chem Biol 9: 174–180. 15811802 10.1016/j.cbpa.2005.02.001

[69] Fukuda H, Hama S, Tamalampudi S, Noda H (2008) Whole-cell biocatalysts for biodiesel fuel production. Trends Biotechnol 26: 668–673. 18976825 10.1016/j.tibtech.2008.08.001

[70] Wohlgemuth R (2009) The locks and keys to industrial biotechnology. New Biotechnol 25: 204–213. 10.1016/j.nbt.2009.01.002 19429540

[71] Xiao Z, Lv C, Gao C, Qin J, Ma C, et al (2010) A novel whole-cell biocatalyst with NAD+regeneration for production of chiral chemicals. PLoS ONE 5: e8860. 20126645 10.1371/journal.pone.0008860 PMC2811184

[72] Yuryev R, Liese A (2010) Biocatalysis: the outcast. ChemCatChem 2: 103–107.

[73] de Carvalho CCCR (2011) Enzymatic and whole cell catalysis: finding new strategies for old processes. Biotechnol Adv 29: 75–83. 20837129 10.1016/j.biotechadv.2010.09.001

[74] Matsuyama A, Yamamoto H, Kobayashi Y (2002) Practical application of recombinant whole-cell biocatalysts for the manufacturing of pharmaceutical intermediates such as chiral alcohols. Org Process Res Dev 6: 558–561.

[75] Gröger H, Rollmann C, Chamouleau F, Sebastien I, May O, et al (2007) Enantioselective reduction of 4-fluoroacetophenone at high substrate concentration using a tailor-made recombinant whole-cell catalyst. Adv Synth Catal 349: 709–712.

[76] Domínguez de María P, Stillger T, Pohl M, Kiesel M, Liese A, et al (2008) Enantioselective C-C bond ligation using recombinant Escherichia coli-whole-cell biocatalysts. Adv Synth Catal 350: 165–173.

[77] Ema T, Ide S, Okita N, Sakaia T (2008) Highly efficient chemoenzymatic synthesis of methyl (*R*)-O-chloromandelate, a key intermediate for clopidogrel, via asymmetric reduction with recombinant Escherichia coli. Adv Synth Catal 350: 2039–2044.

[78] Časar Z (2008) Straightforward and efficient synthesis of (4R,6S)-4-(tert-butyldimethylsiloxy)-6-(hydroxymethyl)tetrahydropyran-2-one. Synlett: 2036–2040.

[79] Časar Z, Tramšek M, Goršek A (2010) Calorimetric insight into coupling between functionalized primary alkyl halide and vinylic organocuprate reagent: experimental determination of reaction enthalpies in the synthesis of (R)-ethyl 3-(tert-butyldimethylsilyloxy)hex-5-enoate – a key lactonized statin side chain precursor. Acta Chim Slov 57: 66–76. 24061657

[80] Troiani V, Cluzeau J, Časar Z (2011) Application of chemoselective pancreatin powder-catalyzed deacetylation reaction in the synthesis of key statin side chain intermediate (4R,6S)-4-(tert-butyldimethylsilyloxy)-6-(hydroxymethyl)tetrahydropyran-2-one. Org Process Res Dev 15: 622–630.

[81] Časar Z, Košmrlj J (2009) The first convenient entry to δ-formyl-δ-valerolactone precursor for the synthesis of statins via lactonized side chain. Synlett: 1144–1148.

[82] Alcaide B, Almendros P (2008) Organocatalytic reactions with acetaldehyde. Angew Chem Int Ed 47: 4632–4634. 10.1002/anie.200801231 18481349

[83] Udovič M, Tramšek M, Plantan I, Cluzeau J (2011) Synthesis of acetoxyacetaldehyde. (Lek Pharmaceuticals d.d.). PCT Int Appl WO 2011064249 A1 Chem Abstr (2011) 154: 617792.

[84] Cluzeau J, Časar Z, Mrak P, Ošlaj M, Kopitar G (2009) ((2S,4R)-4,6-Dihydroxytetrahydro-2H-pyran-2-yl)methyl carboxylate and process for the production thereof. (Lek Pharmaceuticals d.d.). PCT Int Appl WO 2009092702 A2 Chem Abstr (2009) 151: 218948.

[85] DASGIP Technology (2002) DASGIP Application Note on High Density E. coli Fermentation. (DASGIP Information and Process Technology GmbH, an Eppendorf Company) Available: http://www.dasgip.com/media/content/pages/downloads/Application_DASGIP_E.Coli_2002.pdf Accesed 2012 November 15.

[86] Shiloach J, Fass R (2005) Growing E. coli to high cell density–A historical perspective on method development Biotech Adv. 23: 345–357. 10.1016/j.biotechadv.2005.04.004 15899573

[87] Shojaosadati SA, Kolaei SMV, Babaeipour V, Farnoud AM (2008) Recent advances in high cell density cultivation for production of recombinant protein. Iran J Biotech 6: 63–84.

[88] Sharma A, Schulman SG (1999) Introduction to Fluorescence Spectroscopy. New York: Wiley Interscience. 174 p.

[89] Craig EA, Eisenman HC, Hundley HA (2003) Ribosome-tethered molecular chaperones: the first line of defense against protein misfolding? Curr Opin Microbiol 6: 157–162. 12732306 10.1016/s1369-5274(03)00030-4

[90] Tyedmers J, Mogk A, Bukau B (2010) Cellular strategies for controlling protein aggregation. Nat. Rev. Mol. Cell Bio (11) 777–788. 10.1038/nrm2993 20944667

[91] Chiang SM, Schellhorn HE (2012) Regulators of oxidative stress response genes in Escherichia coli and their functional conservation in bacteria. Arch Biochem Biophys 525: 161–169. 22381957 10.1016/j.abb.2012.02.007

[92] Guisbert E, Yura T, Rhodius VA, Gross CA (2008) Convergence of molecular, modeling, and systems approaches for an understanding of the Escherichia coli heat shock response. Microbiol Mol Biol Rev 72: 545–554. 18772288 10.1128/MMBR.00007-08 PMC2546862

[93] Gutsulyak DV, Nikonov GI (2012) Chemoselective Ruthenium-Catalyzed Reduction of Acid Chlorides to Aldehydes with Dimethylphenylsilane. Adv Synth Catal 354: 607–611.

[94] Chuang C-Y, Vassar VC, Ma Z, Geney R, Ojima I (2002) Electronic effects on the regio- and enantioselectivity of the asymmetric aminohydroxylation of O-Substituted 4-hydroxy-2-butenoates. Chirality 14: 151–162. 11835558 10.1002/chir.10050

[95] Crestia D, Guerard C, Bolte J, Demuynck C (2001) Rabbit muscle aldolase (RAMA) as a catalyst in a new approach for the synthesis of 3-deoxy-D-manno-2-octulosonic acid and analogues. J Mol Cat B: Enz 11: 207–212.

[96] Sakuraba H, Yoneda K, Yoshihara K, Satoh K, Kawakami R, et al (2007) Sequential aldol condensation catalyzed by hyperthermophilic 2-deoxy-D-ribose-5-phosphate aldolase. Appl Environ Microbiol 73: 7427–7434. 17905878 10.1128/AEM.01101-07 PMC2168217

[97] Schmidt RR, Zimmermann P (1986) Synthesis of D-erythro-sphingosines. Tetrahedron Lett 27: 481–484.

[98] Wild R, Schmidt RR (1994) Sphingosine and phytosphingosine from D-threose: Synthesis of a 4-keto-ceramide. Tetrahedron: Asymmetry 5: 2195–2208.

[99] lamparuthi E, Ramesh E, Raghunathan R (2012) InCl_3_ as an efficient catalyst for cyclotrimerization of aldehydes. Synthesis of 1,3,5-trioxane under solvent-free conditions. Synth Commun 35: 2801–2804.

[100] Park CP, Lee JH, Yoo KS, Jung KW (2010) Efficient Diacetoxylation of Alkenes via Pd(II)/Pd(IV) Process with Peracetic Acid and Acetic Anhydride. Org Lett 12: 2450–2452. 20455553 10.1021/ol1001686 PMC2880612

[101] O’Connor CJ, Barton RH (1998) Acyl transfer isomerization of Glycerol 1,2-Dibutyrate and Propane-1,2-diol 1-Butyrate. Aust J Chem 51: 455–459.

[102] Steinfeld JI, Francisco JS, Hase WL (1989) Chemical Kinetics and Dynamics. Englewood Cliffs: Prentice Hall. 548 p.

[103] Pravdic N, Fletcher HG (1971) The oxidation of 2-acetamido-2-deoxyaldoses with aqueous bromine. Two diastereoisomeric 2-acetamido-2,3-dideoxyhex-2-enono-1,4-lactones from 2-acetamido-2-deoxy-D-glucose, -mannose, and -gallactose. Carbohydr Res 19: 339–352. 5151898 10.1016/s0008-6215(00)86164-9

[104] Lee T-J (1985) An expeditious chiral route to analogs of mevinolin and compactin. Tetrahedron Lett 26: 4995–4996.

[105] Clive DLJ, Murthy KSK, Wee AGH, Prasad JS, da Silva GVJ, et al (1988) Total synthesis of both (+)-compactin and (+)-mevinolin. A general strategy based on the use of a special TiCl_3_/C_8_K mixture for dicarbonyl coupling. J Am Chem Soc 110: 6914–6916.

[106] Sharma M, Bernacki RJ, Paul B, Korytnyk W (1990) Fluorinated carbohydrates as potential plasma membrane modifiers. Synthesis of 4- and 6-fluoro derivatives of 2-acetamido-2-deoxy-D-hexopyranoses. Carbohydr Res 198: 205–221. 2379186 10.1016/0008-6215(90)84293-4

[107] Rajagopal S, Vancheesan S, Rajaram J, Kuriacose JC (1992) RuCl_2_(PPh_3_)_3_-catalyzed transfer hydrogenation of D-glucose. J Mol Cat 75: 199–208.

[108] Mori Y, Suzuki M (1990) Synthetic study on 1,3-polyols. An efficient enantioselective synthesis of tarchonanthuslactone. J Chem Soc Perkin Trans 1: 1809–1812.

