# Supplementary material for: A Highly Productive, Whole-Cell DERA Chemoenzymatic Process for Production of Key Lactonized Side-Chain Intermediates in Statin Synthesis
Source: PLoS One. 2013 May 7;8(5):e62250. doi: 10.1371/journal.pone.0062250 (PMC3647077; doi:10.1371/journal.pone.0062250)
Supplement: Information S1 — Fermentation data. (PDF) [file pone.0062250.s001.pdf]

Matej Ošlaj,[a] Jérôme Cluzeau,[b] Damir Orkić,[b] Gregor Kopitar,[a] Peter Mrak[a] and Zdenko Časar[b,c]**

Supporting information S1. Fermentation data

Fermentation parameters for a representative process for high-density, DERA-expressing culture of *E. coli* BL21(DE3) pET30/*deoC* is shown below. A representative fermentaion from 70 L Applicon (Netherlands) bioreactors is shown where the protocol described in Materials and Methods was scaled up in a linear manner with starting volume of medium being 27 L.

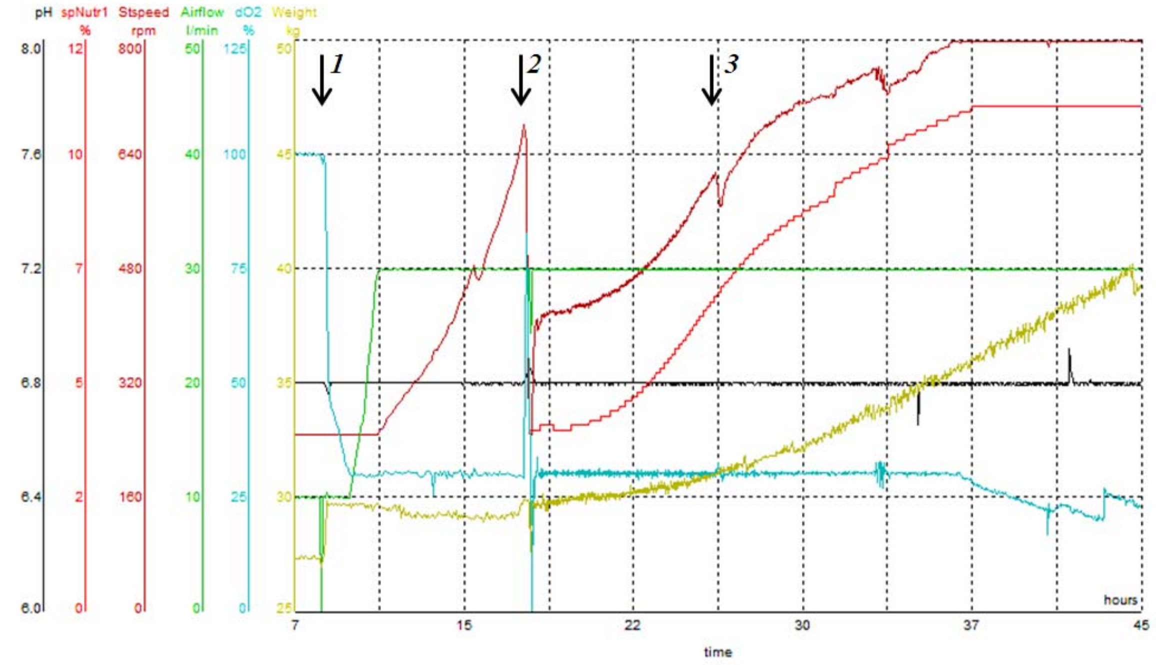

Figure S1A: Fermentation on-line parameters in a representative process for high-density-culture expression of DERA in a 70 L fermenter. Black: pH, red: feeding solution flow rate [%], crimson: stirrer speed [rpm], green: airflow [L min^{-1}], blue: dO_2 [%], yellow: weight [kg]. The process time starts with sterilization therefore the fermentation time starts with inoculation at 8.5 h process time. 1: Inoculation, 2: Depletion of the glycerol, 3: IPTG induction.

The inoculation time was at 8.5 h process time (Figure S1A; 1), and depletion of glycerol in the initial media is well indicated at 18 h process time (10 h after inoculation) in a sharp drop of oxygen consumption and an increase in pH (Figure S1A; 2). From this point on, feeding with the glucose-based feeding solution commences. The induction with IPTG (0.2 mmol L^{-1}) was done at 26 h process time (18 h after inoculation) indicated by a small drop in oxygen consumption, observed in the reduction of stirrer speed enforced by the automatic regulator (Figure S1A; 3). Harvesting commenced at 45 h process time (36.5 h after inoculation). Offline measurements of biomass (WCW) and specific DERA activity are shown in Figure S1B.

A highly productive, whole-cell DERA chemoenzymatic process for production of key lactonized side-chain intermediates in statin synthesis

Supporting information

Matej Ošlaj,[a] Jérôme Cluzeau,[b] Damir Orkić,[b] Gregor Kopitar,[a] Peter Mrak[a] and Zdenko Časar[b,c]**

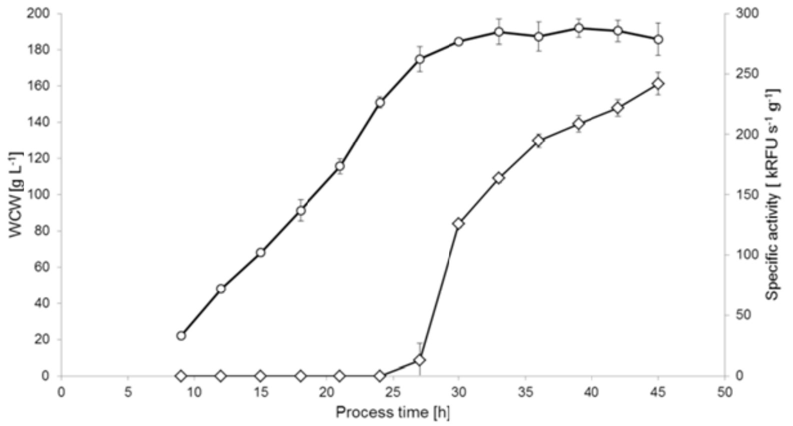

Figure S1B: Fermentation off-line measurements of wet cell weight (WCW) (○) and DERA specific activity of the biomass (◊). Indicated are the analytical errors.

The harvested broth had 180-215 g/L wet cell weight (OD_{600} ~ 100-115), 210-250 kRFU s^{-1} g^{-1} specific DERA activity. The total soluble protein content was 44 mg per g WCW and DERA was found in ~ 50% of the total soluble protein (22 mg per g WCW) as calculated from the Bradford assay and SDS-PAGE analysis of the soluble proteins (Figure S1C).

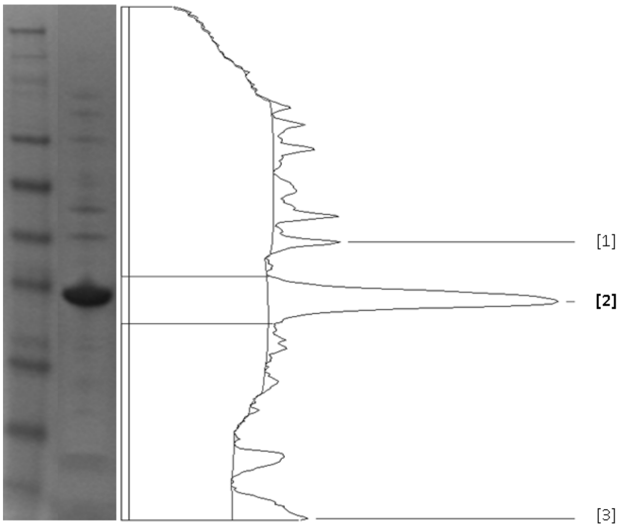

Figure S1C: SDS-PAGE analysis of total soluble protein in the sonicated whole-cell DERA broth. DERA (peak 2) was estimated to represent ~ 50% of total soluble protein.
