# Supplementary material for: A Highly Productive, Whole-Cell DERA Chemoenzymatic Process for Production of Key Lactonized Side-Chain Intermediates in Statin Synthesis
Source: PLoS One. 2013 May 7;8(5):e62250. doi: 10.1371/journal.pone.0062250 (PMC3647077; doi:10.1371/journal.pone.0062250)
Supplement: Information S2 — NMR, HR-MS, GC-FID and GC-MS data. (PDF) [file pone.0062250.s002.pdf]

A highly productive, whole-cell DERA chemoenzymatic process for production of key lactonized side-chain intermediates in statin synthesis

Supporting information

Matej Ošlaj,[a] Jérôme Cluzeau,[b] Damir Orkić,[b] Gregor Kopitar,[a] Peter Mrak[a] and Zdenko Časar[b,c]**

Supporting information S2. NMR, HR-MS, GC-FID and GC-MS data

NMR spectra were recorded on Bruker Avance III spectrometer: 500 MHz for ^{1}H-NMR, Varian VNMRS 400 spectrometer: 400 MHz for ^{1}H-NMR and Varian Unity Inova 300 spectrometer: 300 MHz for ^{1}H-NMR. Chemical shifts are reported in δ ppm referenced to TMS as an internal standard. High-resolution mass spectra (HRMS) were acquired on Agilent 6224 Accurate Mass TOF LC/MS mass spectrometer.

(4*R*,6*R*)-6-methyltetrahydro-2*H*-pyran-2,4-diol (**3a**): (Ma = major anomer, mi = minor anomer)
^{1}H-NMR (500 MHz, DMSO-d$_6$, ppm) δ 6.23 (d, *J* = 6.5 Hz, 1H, Ma), 6.02 (d, *J* = 7.0 Hz, 1H, mi), 5.00 (m, 1H, mi), 4.91 (d, *J* = 5.0 Hz, 1H, mi), 4.85 (m, 1H, Ma), 4.58 (d, *J* = 2.5 Hz, 1H, Ma), 4.21 (m, 1H, mi), 3.83 (m, 1H, Ma), 1.70-1.20 (m, 2H, Ma+mi), 1.06 (d, *J* = 6.0 Hz, 1H, mi), 1.05 (d, *J* = 6.5 Hz, 1H, Ma). ^{13}C-NMR (125 MHz, CDCl$_3$, ppm) δ Major 92.9, 64.9, 59.2, 39.8, 34.9, 21.4, minor 92.3, 66.7, 65.5, 39.4, 39.1, 20.7 HRMS: *m/z* (ESI) calculated $[M + H - H_2O]^+$ 115.0759, measured 115.0755 (Δ = -3.5 ppm). GC-FID response factor (compared to **3g**): 0.753. GC-MS: $[M + H – H_2O]^+$: 115

(4*R*,6*S*)-6-(chloromethyl)tetrahydro-2*H*-pyran-2,4-diol (**3b**):
NMR spectra were found to correspond to those previously described.[57] HRMS: *m/z* (ESI) calculated $[M + H - H_2O]^+$ 149.0369, measured 149.0366 (Δ = -2.2 ppm). GC-FID response factor (compared to **3g**): 0.68. GC-MS: $[M + H – H_2O]^+$: 149

(4*R*,6*S*)-6-((benzyloxy)methyl)tetrahydro-2*H*-pyran-2,4-diol (**3e**) (1:1 mixture of 2 anomers):
^{1}H-NMR (500 MHz, CDCl$_3$, ppm) δ 7.30 (m, 5H), 5.34 (t, *J* = 3.7 Hz, 1H), 5.11 (m, 1H), 5.06 (d, *J* = 5.0 Hz, 1H), 4.74 (d, *J* = 6.5 Hz, 1H), 4.57-4.45 (m, 4H), 4.21-4.09 (m, 3H), 3.89 (d, *J* = 7.2 Hz, 1H), 3.53-3.41 (m, 4H), 2.57 (br s, 1H), 2.47 (br s, 1H), 1.93-1.40 (m, 8H). ^{13}C-NMR (125 MHz, CDCl$_3$, ppm) δ 137.7, 128.3, 127.7, 127.6, 92.6, 92.1, 73.3, 73.1, 72.9, 69.6, 64.8, 64.3, 62.4, 39.2, 34.9, 34.2, 33.9. HRMS: *m/z* (ESI) calculated $[M + H - H_2O]^+$ 221.1178, measured 221.1169 (Δ = -3,9 ppm). GC-FID response factor (compared to **3g**): 1.31. GC-MS: $[M + H - 2H_2O]^+$: 203

(4*R*,6*S*)-6-(dimethoxymethyl)tetrahydro-2*H*-pyran-2,4-diol (**3f**):
^{1}H-NMR (300 MHz, CDCl$_3$): δ = 5.25 (d), 4.35-4.15 (m), 3.42 (s), 1.9-1.4 (m).. ^{13}C-NMR (75 MHz, CDCl$_3$): δ = 105.7, 92.9, 64.5, 62.8, 54.9, 54.5, 35.0, 32.8. . HRMS: *m/z* (ESI) calculated $[M + H - H_2O]^+$ 175.0970, measured 175.0973 (Δ = 1.5 ppm). GC-FID response factor (compared to **3g**): 0.92. GC-MS: $[M + H - H_2O]^+$: 175

Matej Ošlaj,[a] Jérôme Cluzeau,[b] Damir Orkić,[b] Gregor Kopitar,[a] Peter Mrak[a] and Zdenko Časar[b,c]**

((2*S*,4*R*)-4,6-dihydroxytetrahydro-2*H*-pyran-2-yl)methyl acetate (**3g**) (major isomer):

^{1}H-NMR (500 MHz, $CDCl_3$ + 0.1% CD_3OD, ppm) δ 5.38 (br s, 1H), 4.53 (m, 1H), 4.30 (m, 1H), 4.14 (m, 2H), 2.11 (s, 3H), 2.05 (m, 1H), 1.88-1.62 (m, 3H). ^{13}C-NMR (125 MHz, DMSO-d_6) δ 170.8, 91.7, 68.1, 66.8, 63.3, 33.9, 20.7. HRMS: *m/z* (ESI) calculated $[M + H - H_2O]^+$ 173.0814, measured 173.0810 (Δ = -2.2 ppm). GC-MS: $[M + H – H_2O]^+$: 173

((4*R*,6*R*)-4-hydroxy-6-methyltetrahydro-2*H*-pyran-2-one (**15a**):

^{1}H-NMR (500 MHz, acetone-d_6) δ 4.76 (dq, J_d = 11.2 Hz, J_q = 3.2 Hz, 1H), 4.41-4.15 (m, 2H), 2.63 (dd, *J* = 4.3 Hz, *J* = 17.0 Hz, 1H), 2.46 (ddd, *J* = 1.7 Hz, *J* = 3.3 Hz, *J* = 17.0 Hz, 1H), 1.92 (m, 1H), 1.71 (dd, *J* = 3.0 Hz, *J* = 14.3 Hz, 1H), 1.29 (d, *J* = 6.4 Hz, 3H). ^{13}C-NMR (125 MHz, acetone-d_6) δ 170.6, 72.7, 63.1, 39.1, 38.2, 21.8. HRMS: m/z (ESI) calculated $[M + H]^+$ 131.0703, measured 131.0702 (Δ = -0.5 ppm). GC-MS: $[M + H]^+$: 131

((2*S*,4*R*)-4-hydroxy-6-oxotetrahydro-2*H*-pyran-2-yl)methyl acetate (**15g**):

^{1}H-NMR (300 MHz, acetone-d_6) δ 4.88 (m, 1H), 4.45 (d, *J* = 3.0 Hz, 1H), 4.38 (hex, *J* = 3.0 Hz, 1H), 4.23 (dd, *J* = 3.5 Hz, *J* = 12.0 Hz, 1H), 4.16 (dd, *J* = 5.5 Hz, *J* = 12.1 Hz, 1H), 2.68 (dd, *J* = 4.3 Hz, *J* = 17.5 Hz, 1H), 2.51 (dddd, *J* = 0.8 Hz, *J* = 2.0 Hz, *J* = 3.3 Hz, *J* = 17.5 Hz, 1H), 2.03 (s, 3H), 1.91 (m, 2H). ^{13}C-NMR (75 MHz, acetone-d_6) δ 170.8, 169.7, 74.2, 66.5, 62.7, 39.1, 32.3, 20.6. HRMS: *m/z* (ESI) calculated $[M + H]^+$ 189.0757, measured 189.0760 (Δ = 1.3 ppm). GC-MS: $[M + H]^+$: 188
