# Supplementary material for: A Highly Productive, Whole-Cell DERA Chemoenzymatic Process for Production of Key Lactonized Side-Chain Intermediates in Statin Synthesis
Source: PLoS One. 2013 May 7;8(5):e62250. doi: 10.1371/journal.pone.0062250 (PMC3647077; doi:10.1371/journal.pone.0062250)
Supplement: Information S3 — NMR, HR-MS, GC-FID and GC-MS identification of the mono-aldol intermediates (8, 9, 12). (PDF) [file pone.0062250.s003.pdf]

Matej Ošlaj,[a] Jérôme Cluzeau,[b] Damir Orkić,[b] Gregor Kopitar,[a] Peter Mrak[a] and Zdenko Časar[b,c]**

Supporting information S3. NMR, HR-MS, GC-FID and GC-MS identification of the mono-aldol intermediates (8, 9, 12)

GC-MS and HR-MS (where available) data showed a major m/z present for the mono-aldol intermediates:

8a: Not found.

8b: HRMS (ESI+) *m/z* (ESI) calculated $[M + H]^+$ 123.0207 measured 123.0206 (Δ = -1.1 ppm). GC-FID response factor (compared to **3g**): 0.55. GC-MS: $[M + H – H_2O]^+$: 105, $[M + H]^+$: 123

8e: GC-MS: $[M + H - 2H_2O]^+$:159, $[M + H - H_2O]^+$: 177

8f: GC-MS: $[M + H - 2H_2O]^+$:117, $[M + H - H_2O]^+$: 130

8g: HRMS (ESI+) m/z (ESI) calculated [M + H]+ 147.0652, measured 147.0651, (Δ = -0.6 ppm). GC-FID response factor (compared to **3g**): 0.867. GC-MS: $[M + H – H_2O]^+$: 129

^{1}H-NMR identification of the purified compound **8g** proved difficult. Although a single peak (85% area, 10% of aldehyde 2g is the major impurity) can be observed in GC-FID and GC-MS, the compound is present in the samples as a mixture of its aldehyde form, hydrate form **12** and several complex acetal / hemiacetal structures **9** which are difficult to identify. The observation of a single peak on the GC analysis is due to high temperature and dehydrating conditions during the analysis which presumably results in the shift of equilibrium strongly in the direction of the aldehyde form, and is not indicative of the true state of the equilibrium as observed at the reaction conditions by ^{1}H-NMR measurements. Therefore all of the measurements performed by GC analysis show a sum of the equilibrium foms.

The structures of the acetal/hemiacetal forms are difficult to solve due to the following reasons; acetal/hemiacetal form **9** in not a single compound / isomer, the species **8**, **9** and **12** are in equilibrium with each other and the ratio between the various species is changing depending on solvent, water content in the solvent, pH, temerature and the time of preparation of the NMR sample.

Existence of dimeric lactols has been proposed for monoaldol condensation products of other hydroxyl-aldehydes before.[42] The ^{1}H-NMR spectra of the purified **8g,** show at least 3 distinct, acetal specific signals in addition to easily distinguishable **8g** and**12g.** Therefore, we assume the possible presence of other cyclic acetals or hemiacetals. According to chemical knowledge and published examples, the species **9** may include **cyclic lactols**[42] (2 possible anomers), **dimeric lactols** (4 possible isomers)[97], **dimeric hemiacetals** (4 possible isomers)[98] or **cyclic trioxane** (8 possible isomers)[99]. (Figure S3A).

Matej Ošlaj,[a] Jérôme Cluzeau,[b] Damir Orkić,[b] Gregor Kopitar,[a] Peter Mrak[a] and Zdenko Časar[b,c]**

Interestingly, (*S*)-2-hydroxy-4-oxobutyl acetate (**18**) was isolated as a side product after the chemical oxidation. The GC-MS and ^{1}H-NMR data for this compound were in accordance with the literature.[101] Product (**18**) presumably originates from the rearranged mono-aldol condensation product (**16**) *via* its cyclic hemiacetal form (**17**).

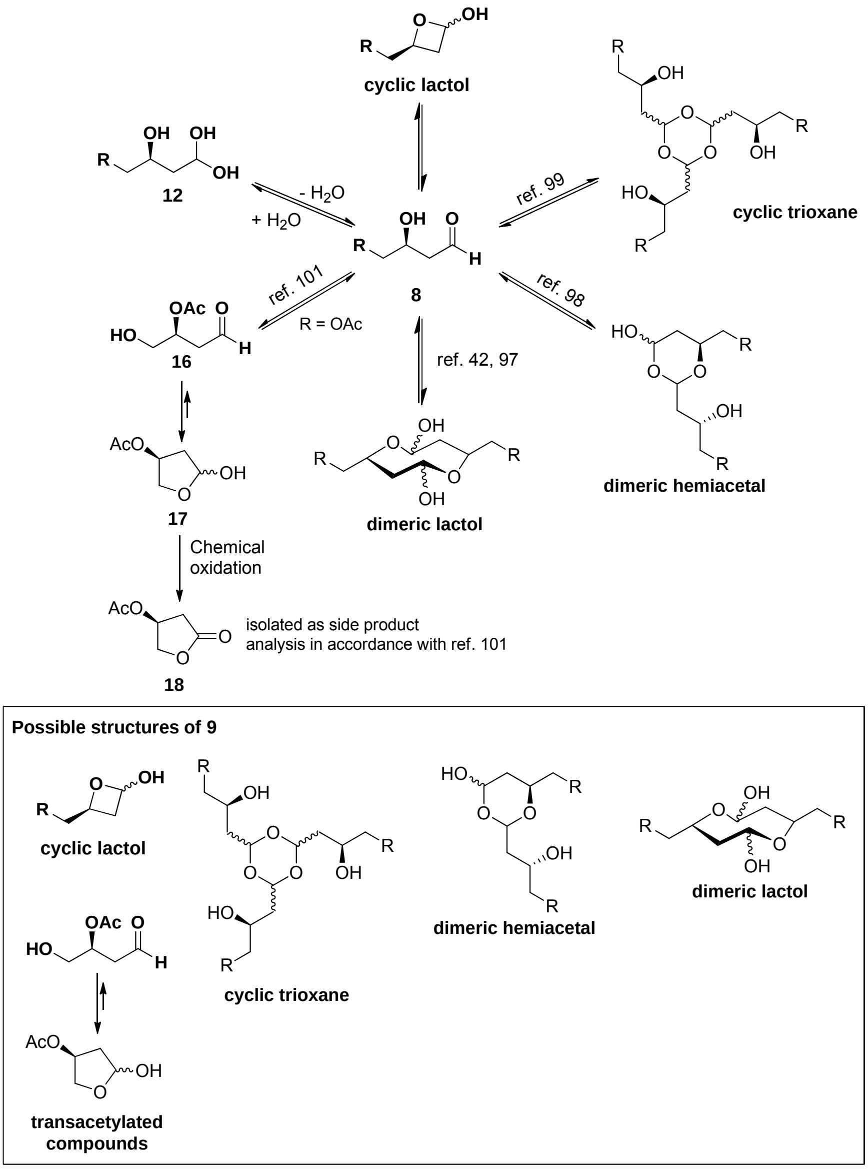

Figure S3A. Possible acetals / hemiacetals arising from the DERA single condensation product **8**. For references see the main article.

In $CDCl_3$: isolated compound **8g** (> 85% purity by GC analysis + 10% aldehyde **2g**) can be well characterized by acquisition of ^{1}H-NMR in $CDCl_3$ right after dissolution (Figure S3B); specific signals: 9.85 (s, 1H), 4.43 (m, 1H), 2.70 (m, 2H). The sample also contains some amount of hydrate **12g** and other unidentified hemiacetals or acetals. When the sample is settled for few days in chloroform, the phase separation between water and chloroform over the time leads to a drier sample. This drier contains has significantly less hydrate **12g** and should contain significantly higher amounts of the aldehyde **8g.** This dehydration of aldehyde hydrate in chloroform was previously shown on similar aldehydes.[81] ^{1}H NMR spectra (figure S3B),however, showed that aldehyde **8g** is decreasing in favor of new compounds having acetal proton signals between 4.7 and 5.6 ppm.

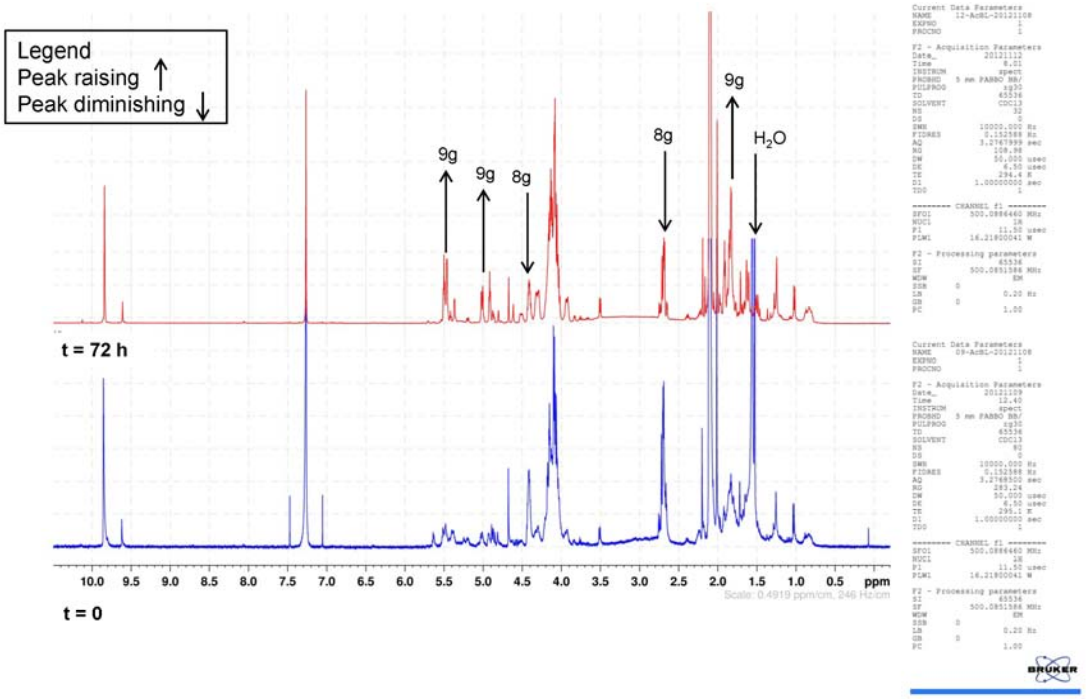

Figure S3B. ^{1}H-NMR comparison after **8g** sample dissolution in $CDCl_3$ (t = 0, blue color) and after 3 days settling (t = 72 h, red color). Changes are indicated by arrows (increasing of compound level or decreasing of compound level).

Matej Oślaj,[a] Jérôme Cluzeau,[b] Damir Orkić,[b] Gregor Kopitar,[a] Peter Mrak[a] and Zdenko Časar[b,c]**

In D_2O: when isolated aldehyde **8g** is dissolved in D_2O, easily identifiable hydrate **12g** (dd at 5.10 ppm) was obtained as the major product. Some aldehyde **8g** is also present (m at 2.65 ppm and s at 9.56 ppm) as well as at least 3 other compounds (m at 5.30 ppm, d at 5.00 ppm and t at 4.82 ppm in respective ratio 80/130/100) again indicating presence of acetals **9**. Presumably the cyclic hemiacetal is the major acetal present. The ratio between the different species is changing with the concentration of the sample (Figure S3C).

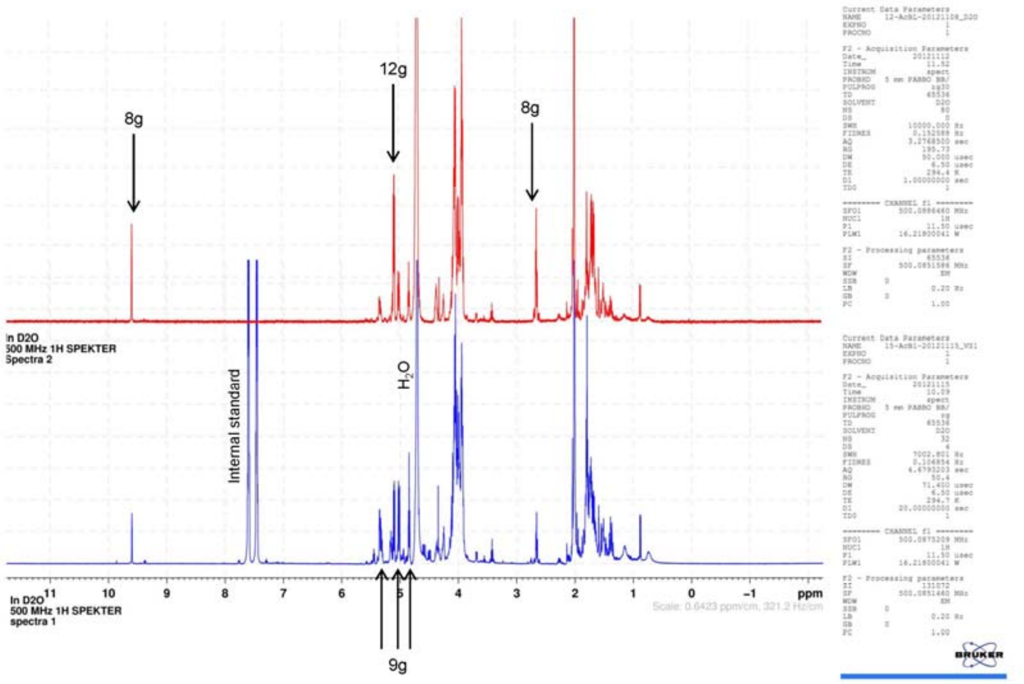

Figure S3C. ^{1}H-NMR spectra comparison of compound **8g** in D_2O at 2 different concentrations (low concentration in red and high concentration (×2.5) in blue). Changes are indicated by arrows (increasing of compound level or decreasing of compound level).

In DMSO-d_6: when isolated aldehyde **8g** is dissolved in DMSO-d_6, a very complex mixture of compounds was obtained with only low amount (about 6%) of free aldehyde **8g** observed in the mixture.
