# Supplementary material for: A Highly Productive, Whole-Cell DERA Chemoenzymatic Process for Production of Key Lactonized Side-Chain Intermediates in Statin Synthesis
Source: PLoS One. 2013 May 7;8(5):e62250. doi: 10.1371/journal.pone.0062250 (PMC3647077; doi:10.1371/journal.pone.0062250)
Supplement: Information S4 — Proton NMR of aldehyde precursors 1 and 2g in D2O. (PDF) [file pone.0062250.s004.pdf]

A highly productive, whole-cell DERA chemoenzymatic process for production of key lactonized side-chain intermediates in statin synthesis

Supporting information

Matej Ošlaj,[a] Jérôme Cluzeau,[b] Damir Orkić,[b] Gregor Kopitar,[a] Peter Mrak[a] and Zdenko Časar[b,c]**

Supporting information S4. Proton NMR of aldehyde precursors 1 and 2g in D_2O

^{1}H-NMR of initial substrates **1** and **2g** shows that they are found in D_2O only in equilibrium between hydrate and aldehyde forms. Interestingly, the acetyloxy-acetaldehyde (**2g**) is found predominantly in the form of hydrate, making accessibility of the reactive aldehyde (**2g**) form even lower compared to acetaldehyde (**1**). (Figure S4A and S4B) This is another indicator which should be considered when interpreting observed reaction dynamics in terms of substrate availability.

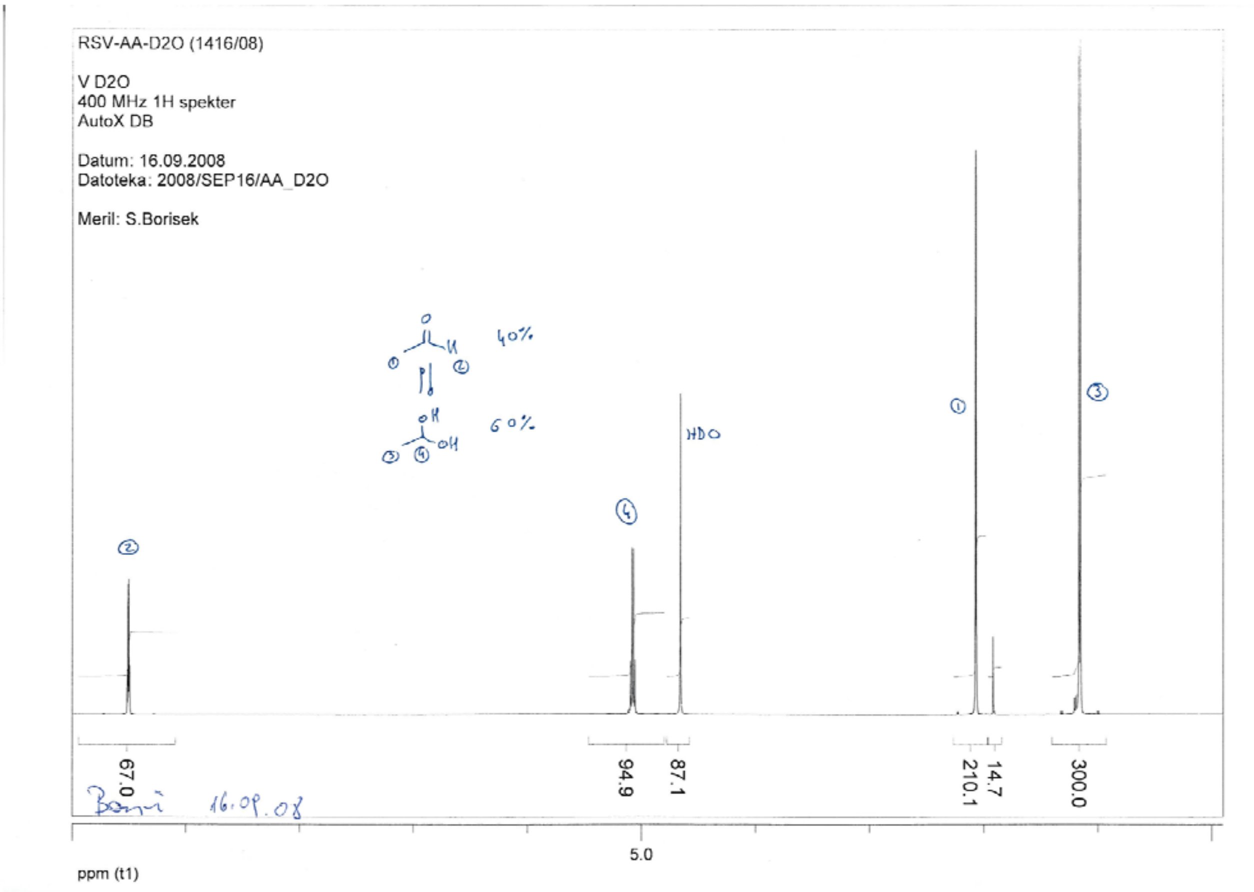

Figure S4A. ^{1}H-NMR spectra of acetaldehyde **1** in D_2O showing mixture of aldehyde **1** and its hydrate form.

Matej Oślaj,[a] Jérôme Cluzeau,[b] Damir Orkić,[b] Gregor Kopitar,[a] Peter Mrak[a] and Zdenko Časar[b,c]**

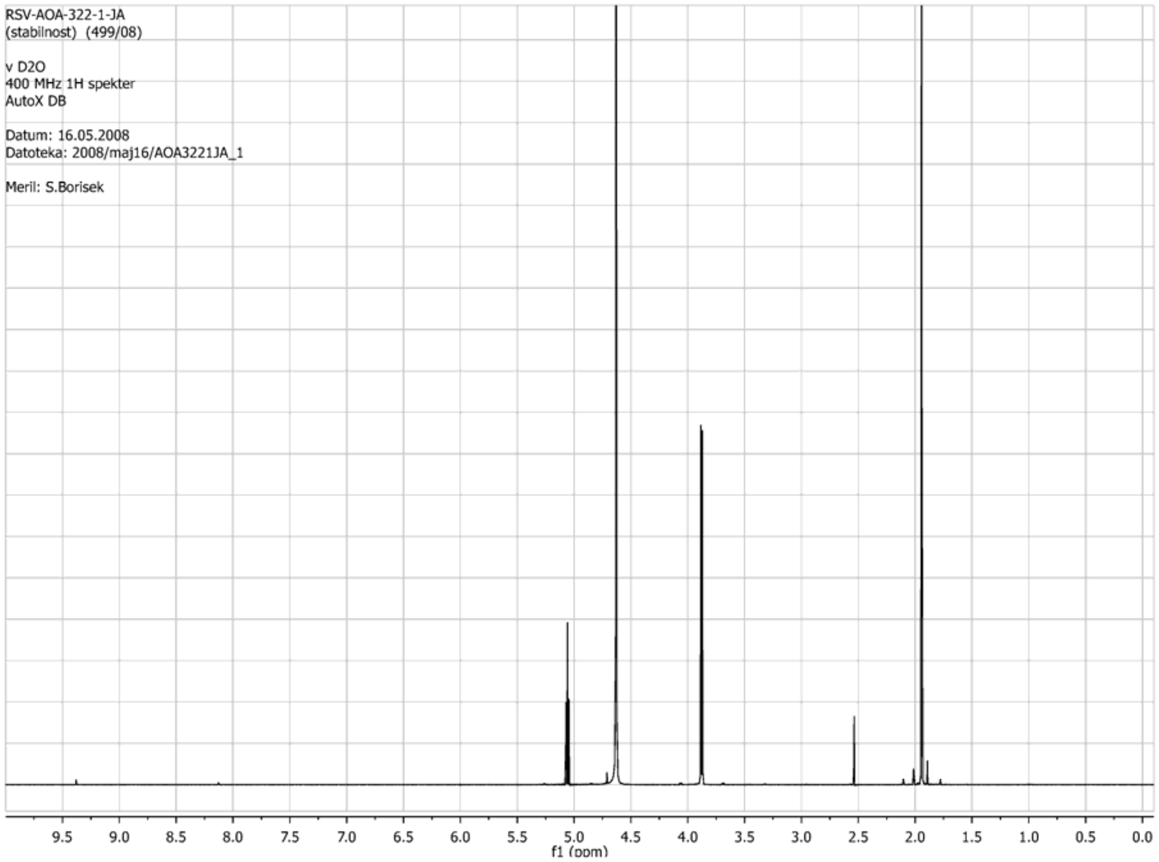

Figure S4B. ^{1}H-NMR spectra of **2g** in D_2O showing mixture of aldehyde **2g** and its hydrate form.
