# Supplementary material for: A Highly Productive, Whole-Cell DERA Chemoenzymatic Process for Production of Key Lactonized Side-Chain Intermediates in Statin Synthesis
Source: PLoS One. 2013 May 7;8(5):e62250. doi: 10.1371/journal.pone.0062250 (PMC3647077; doi:10.1371/journal.pone.0062250)
Supplement: Information S5 — Determination of stereochemical purity. (PDF) [file pone.0062250.s005.pdf]

Matej Ošlaj,[a] Jérôme Cluzeau,[b] Damir Orkić,[b] Gregor Kopitar,[a] Peter Mrak[a] and Zdenko Časar[b,c]**

Supporting information S5. Determination of stereochemical purity

Due to stability issues and unfavorable physical properties of lactols **3**, the stereochemical purity could be determined only by their conversion to known advanced statin intermediates like (4*R*,6*S*)-4-(*tert*-butyldimethylsilyloxy)-6-(hydroxylmethyl)tetrahydropyran-2-one **6**.[78] Therefore, lactols **3** were first oxidized to lactones **15** followed by protection of hydroxyl group on 4-postion to give protected derivatives **5**. The acetyl cleavage[78,80] gave well know alcohol **6**, which is suitable for determination of stereochemical purity by various analytical methods.

((2*S*,4*R*)-4-(*tert*-butyldimethylsilyloxy)-6-oxotetrahydro-2*H*-pyran-2-yl)methyl acetate (**5**).[78]

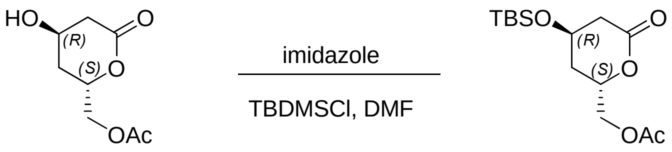

15g **5**

The solution of ((2*S*,4*R*)-4-hydroxy-6-oxotetrahydro-2*H*-pyran-2-yl)methyl acetate (**15g**) (1.88 g, 10 mmol, 1 eq) was dissolved in dry DMF (2 mL, 1M). Imidazole (0.88 g, 1.3 eq) and TBDMSCl (1.66 g, 1.1 eq) were successively added and the reaction was stirred until the completion of the reaction. The reaction mixture was partitioned between water (20 mL) and ether (20 mL). The aqueous phase was extracted once with ether (20 mL). The combined organic phases were washed twice with a small amount of water (10 mL), with HCl 1N (20 mL) and with brine (20 mL). The solution was dried over $MgSO_4$ and concentrated to furnish ((2S,4R)-4-(tert-butyldimethylsilyloxy)-6-oxotetrahydro-2H-pyran-2-yl)methyl acetate (**5**) in a quantitative yield. This is a known compound with spectroscopic and physical properties in accordance with those reported in the literature.[78] ^{1}H NMR (300 MHz, $CDCl_3$) δ 4.93 (m, 1H), 4.37 (quint, *J* = 3 Hz, 1H), 4.30 (dd, *J* = 3 Hz, *J* = 12 Hz, 1H), 4.21 (dd, *J* = 5 Hz, *J* = 12 Hz, 1H), 2.62 (d, *J* = 4 Hz, 2H), 2.11 (s, 3H), 1.84-1.80 (m, 2H), 0.89 (s, 9H), 0.09 (2s, 6H). ^{13}C NMR (75 MHz, $CDCl_3$) δ 170.4, 169.1, 73.3, 65.5, 63.0, 38.9, 32.2, 20.5, 17.7, -5.1, -5.2.

Matej Ošlaj,[a] Jérôme Cluzeau,[b] Damir Orkić,[b] Gregor Kopitar,[a] Peter Mrak[a] and Zdenko Časar[b,c]**

(4*R*,6*S*)-4-(*tert*-butyldimethylsilyloxy)-6-(hydroxymethyl)tetrahydro-2*H*-pyran-2-one (**6**).[80]

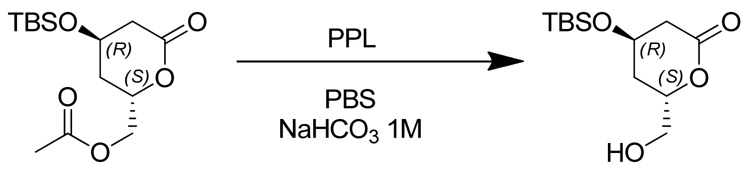

5 **6**

((2*S*,4*R*)-4-(*tert*-butyldimethylsilyloxy)-6-oxotetrahydro-2*H*-pyran-2-yl)methyl acetate (**5**) (50 g, 80% purity; 132,4 mmol) was added to phosphate buffer solution (P.B.S.) pH=5,20 (1,5 L), the solution was warmed up to 37°C. Pancreatin powder (Porcine pancreatic lipase, PPL, 0,5 eq. mass; 20 g) was then added stepwise (6 times, 8 g + 3 x 4 g). In parallel, pH was monitored and was regulated by adding $NaHCO_3$ solution (1M) each hour to maintain pH between 4.85 and 4.95. The reaction was stirred for 9 hours after the first addition of enzyme. Celite® was added to the crude mixture. The solution was filtered off through celite® and a pale yellow liquid was recovered. The solid on the filter was washed with 1.5 L of EtOAc. The filtrate was stirred for 5 minutes followed by separation of the two layers. The water phase was re-extracted once with EtOAc (1.5 L). The combined organic phases were partially evaporated under reduced pressure at 40 °C and the product was recrystallized from methylcyclohexan to give 82% of (4*R*,6*S*)-4-(*tert*-butyldimethylsilyloxy)-6-(hydroxymethyl)tetrahydro-2*H*-pyran-2-one (**6**) as white crystals. This is a known compound with spectroscopic and physical properties in accordance with those reported in the literature.[78,80] GC analysis for determination of enantiomeric purity was done with a DCM solution (2-3 mg/mL) on a Betadex 120 column with a split/splitless injector and a FID detector and a single enantiomer was detected (detection limit 0.1%). NMR analysis of compound (**6**) showed absence of diastereomers even with high scan accumulation at detection limit, which indicates that the level of diastereomers in (**6**) is below 0.5% %.

Deprotection of compound (**5**) with ($t$$Bu_2SnClOH)_2$ catalyst[78] provided compound (**6**) which demonstrated the same purity as the one obtained from enzymatic deprotection.

Standards of the enantiomer and diastereomers were prepared according to our previously published procedure based on iodolactonization reaction starting from (*S*)-ethyl 4-chloro-3-hydroxybutanoate or (*R*)-ethyl 4-chloro-3-hydroxybutanoate.[78] Taken together, the results show excellent enantiomeric and diastereomeric purities (>99.8% ee, >99,5de) presumably originating from aldolase catalysed conversion of **2g** to **3g**.
