# Supplementary material for: A Highly Productive, Whole-Cell DERA Chemoenzymatic Process for Production of Key Lactonized Side-Chain Intermediates in Statin Synthesis
Source: PLoS One. 2013 May 7;8(5):e62250. doi: 10.1371/journal.pone.0062250 (PMC3647077; doi:10.1371/journal.pone.0062250)
Supplement: Information S6 — Comparison of DERA activity in the whole broth, washed cells and cell-free lysate with the assay mixture supernatant. DERA assay on whole cells. (PDF) [file pone.0062250.s006.pdf]

Matej Ošlaj,[a] Jérôme Cluzeau,[b] Damir Orkić,[b] Gregor Kopitar,[a] Peter Mrak[a] and Zdenko Časar[b,c]**

Supporting information S6.

A: Comparison of DERA activity in the whole broth, washed cells and cell-free lysate with the assay-mixture supernatant

The activity of freshly harvested whole-cell broth (whole broth, washed cells, culture supernatant and whole-broth lysate) was measured using the 7-deoxyribosyl-4-methyl umbelliferone assay as described in the methods section. The activity data measured were: 230 ± 3 kRFU s^{-1} g^{-1} for the whole broth, 210 ± 5 kRFU s^{-1} g^{-1} for the washed cells, 10.4 ± 4.35 kRFU s^{-1} g^{-1} for the culture supernatant and 233 ± 6 kRFU s^{-1} g^{-1} for the cell-free lysate of the culture broth.

B. DERA assay on whole cells

In order to study whether intracellular DERA can catalyze the retroaldol reaction on the 7-deoxyribosyl-4-methyl umbelliferone one modification was made to the assay protocol. The 100 mM Bis-Tris propane, pH 8.5 buffer was replaced with PBS in order to reduce partial lysis of the cells induced by the combination of high pH and osmotic stress caused by the former buffer. The lower pH of the PBS (pH = 7.0) decreases fluorescence efficiency of 4-methyl umbelliferone about 4 fold, therefore these conditions were used for the purpose of this experiment only.

The activity data measured with the modified assay were: 40.0 ± 3.2 kRFU s^{-1} g^{-1} for the whole broth, 37.5 ± 0.5 kRFU s^{-1} g^{-1} for the washed cells, 2.1 ± 0.07 kRFU s^{-1} g^{-1} for the culture supernatant and 52.0 ± 2.2 kRFU s^{-1} g^{-1} for the cell-free lysate of the culture broth. Upon completion of the assay (60 min), samples were centrifuged and analyzed with SDS-PAGE for presence of DERA in the assay supernatants. Due to high dilution of assay samples, silver stain was used as a contrasting agent (Figure S6). The release of DERA from the cells in this experiment was minimal and the end point fluorescence for the whole-cell assay mixture and the whole-cell assay supernatant was at the same level.

Matej Oślaj,[a] Jérôme Cluzeau,[b] Damir Orkić,[b] Gregor Kopitar,[a] Peter Mrak[a] and Zdenko Časar[b,c]**

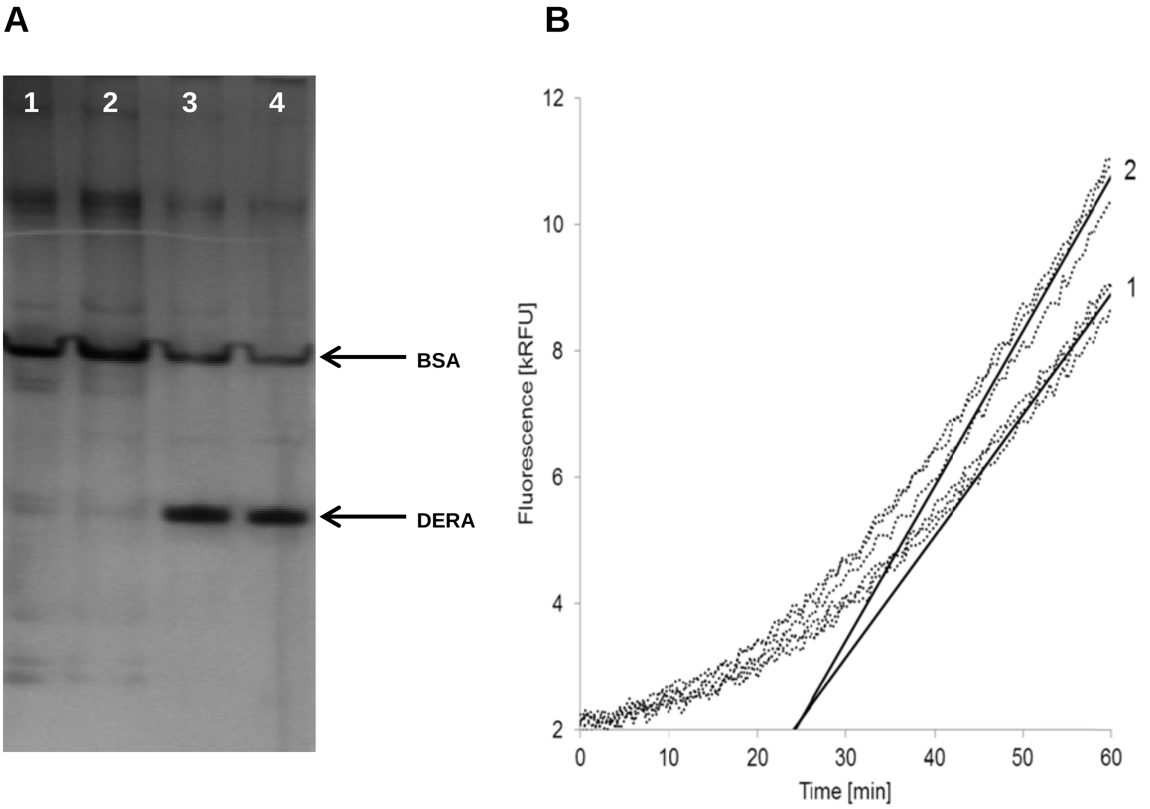

Figure S6: Silver stain SDS-PAGE of the DERA activity assay supernatants and assay fluorescence raw data. A: Silver stain SDS-PAGE gel. Lanes: washed cells before the assay (1), washed cells after the assay (2), cell-free lysate before the assay (3), cell-free lysate after the assay (4). B: DERA activity assay raw data. Washed cells (1): specific DERA activity 37.5 ± 0.5 kRFU s^{-1} g^{-1}, cell-free lysate (2): specific DERA activity 52.0 ± 2.2 kRFU s^{-1} g^{-1}.
