# Supplementary material for: A Highly Productive, Whole-Cell DERA Chemoenzymatic Process for Production of Key Lactonized Side-Chain Intermediates in Statin Synthesis
Source: PLoS One. 2013 May 7;8(5):e62250. doi: 10.1371/journal.pone.0062250 (PMC3647077; doi:10.1371/journal.pone.0062250)
Supplement: Information S7 — Residual DERA activity measurements and DERA distribution in the whole cell process with 2g. (PDF) [file pone.0062250.s007.pdf]

Matej Ošlaj,[a] Jérôme Cluzeau,[b] Damir Orkić,[b] Gregor Kopitar,[a] Peter Mrak[a] and Zdenko Časar[b,c]**

Supporting information S7. Residual DERA activity measurements and DERA distribution in the whole cell process with 2g.

Comparing the whole-cell-catalyzed *E. coli* BL21 (DE3) pET30/*DeoC* fed-batch reaction (400mM 2g, 840mM acetaldehyde) with parallel reaction, catalyzed with the DERA cell-free lysate, we observed no differences in reaction-species dynamics or yields. Both reactions were set up to have equal initial volumetric activity of DERA (38419.2 kRFU s^{-1} L^{-1}). In order to confirm that the DERA-catalyzed coupling of the substrate aldehydes takes place intracellularly, we sampled the whole-cell reaction and compared the amount of DERA present in washed cells to the amount of DERA found in the reaction supernatant (Figure S7). The slight increase in the amount of the extracellular DERA protein (detected on the SDS-PAGE gels) indicates partial lysis of the cells during the reaction. Compared to the cell-free lysate reaction samples, the quantity of the DERA released extracellularly is so small that it can hardly influence the reaction flow.

A **B**

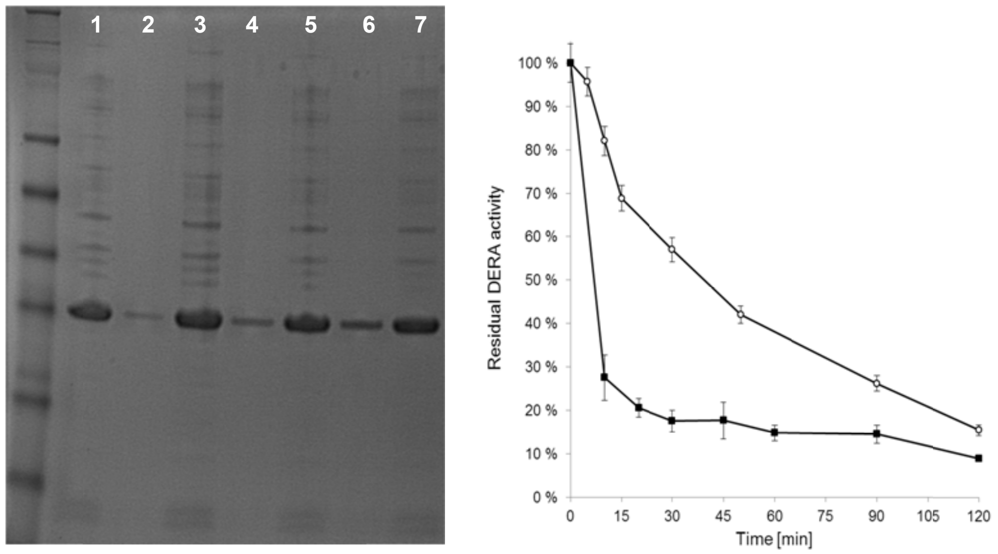

Figure S7: Distribution of DERA protein and residual activity during whole-cell catalyst and cell-free lysate reaction. **A**: SDS-PAGE showing distribution of DERA in a whole-cell, batch reactions using 400mM of **2g** and 840mM of acetaldehyde. Soluble protein from the reaction supernatant at; 0 min (lane 2), 30 min (lane 4) and 60 min (lane 6) in the whole- cell reaction. Soluble protein from the washed cells at; 0 min (lane 3), 30 min (lane 5) and 60 min (lane 7). The soluble protein in the reaction supernatant using cell-free lysate as the catalyst at 0 min is shown in lane 1. **B:** Comparison of residual DERA activity in batch (■) and fed-batch (○) process mode using 400mM of **2g** and 840mM of acetaldehyde. The fluorimetric activity assay described in the methods section was used for determination of DERA activity.

Matej Ošlaj,[a] Jérôme Cluzeau,[b] Damir Orkić,[b] Gregor Kopitar,[a] Peter Mrak[a] and Zdenko Časar[b,c]**

In addition, residual activity of the washed cell samples in the above whole-cell-catalyzed fed-batch reaction was compared to the residual activity of the washed biomass, sampled from the batch reaction (main article, figure 5). The same DERA whole-cell catalyst having DERA specific activity 232 kRFU s-1 g-1, WCW = 207 g L-1 was used in both experiments. The specific DERA activity measured by the fluorometric assay is shown in figure SP7. The results show big differences in the residual DERA activity profile in favor of the fed batch process.
