# Supplementary material for: A Highly Productive, Whole-Cell DERA Chemoenzymatic Process for Production of Key Lactonized Side-Chain Intermediates in Statin Synthesis
Source: PLoS One. 2013 May 7;8(5):e62250. doi: 10.1371/journal.pone.0062250 (PMC3647077; doi:10.1371/journal.pone.0062250)
Supplement: Information S8 — Proposed alternative double condensation product observed in reactions with 2b. (PDF) [file pone.0062250.s008.pdf]

*Matej Ošlaj,[a] Jérôme Cluzeau,[b] Damir Orkić,[b] Gregor Kopitar,[a] Peter Mrak[a]* and Zdenko Časar[b,c]**

**Supporting information S8. Proposed alternative double condensation product observed in reactions with 2b.**

A significant amount of a DERA-derived product was found accumulating in the batch reactions using 400 mmol $L^{-1}$ of **2b** and 840 mmol $L^{-1}$ of acetaldehyde. Two chromatographically separated isomers having identical mass spectra were observed in the GC-MS analysis (Figure S9). The mass spectra correspond to the lactol formed by aldol coupling of the single aldol intermediate **8b** with additional molecule of **2b** (2,6-chloro-2,4-dideoxyhexose**,** (4R,6S)-3-chloro-6-(chloromethyl)tetrahydro-2H-pyran-2,4-diol). The isotope distribution clearly shows the presence of two chlorine atoms in the compound (Figure SP9). Comparison of the mass spectra for **3b** and **19**, shows characteristic fragments: $[M - H2O + H]^+$, $[M - 2 H_2O + H]^+$ and $[M - H_2O - HCl + H]^+$. We have not observed any single aldol condensation products having two chlorine atoms accumulating in the reactions, therefore we propose the origin of this molecule as the product of the DERA-catalyzed coupling of **8b** with alcetaldehyde as depicted in figure S8.

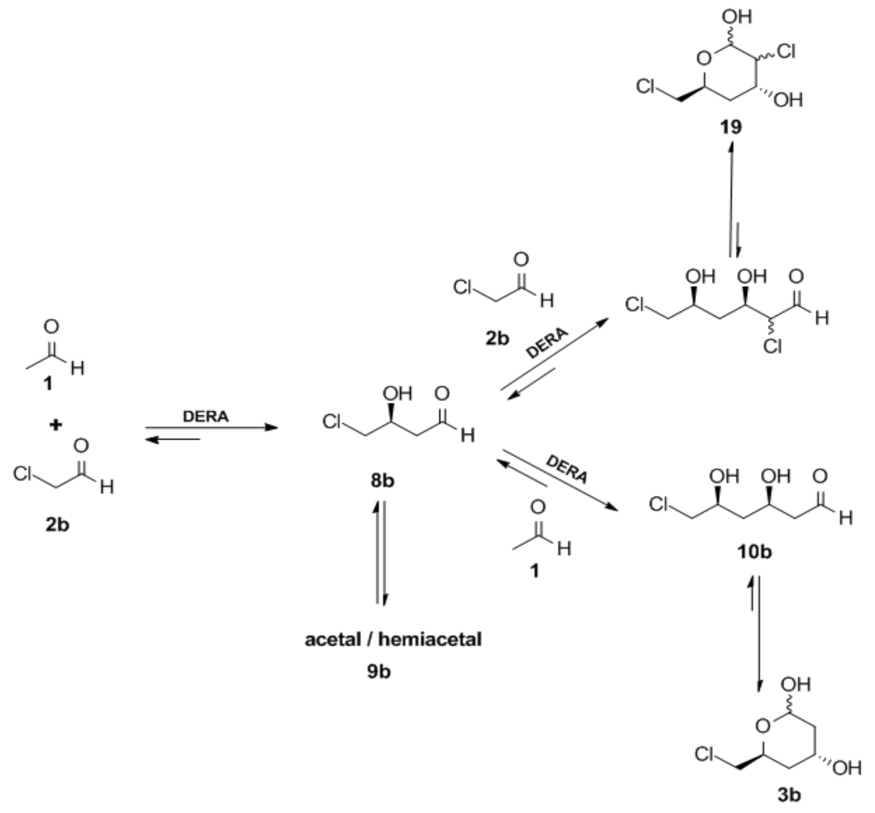


**Figure S8: Proposed reaction species in DERA condensation of 2b and 1.**

A highly productive, whole-cell DERA chemoenzymatic process for production of key lactonized side-chain intermediates in statin synthesis

Supporting information

*Matej Ošlaj,[a] Jérôme Cluzeau,[b] Damir Orkić,[b] Gregor Kopitar,[a] Peter Mrak[a]* and Zdenko Časar[b,c]**

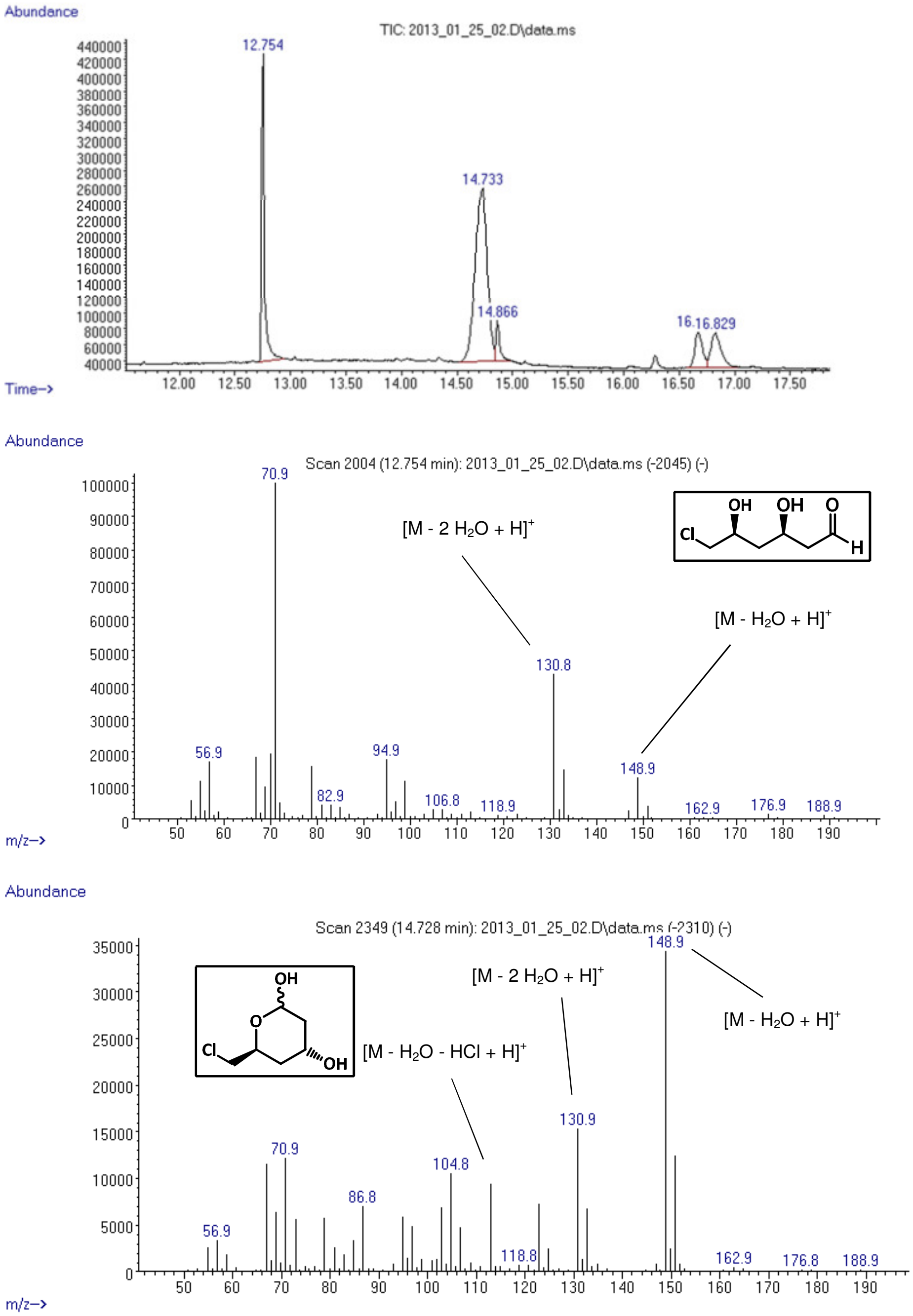


**Figure S9 part 1: GC-MS spectra indicating the structure of 19.**

*Matej Ošlaj,[a] Jérôme Cluzeau,[b] Damir Orkić,[b] Gregor Kopitar,[a] Peter Mrak[a]* and Zdenko Časar[b,c]**

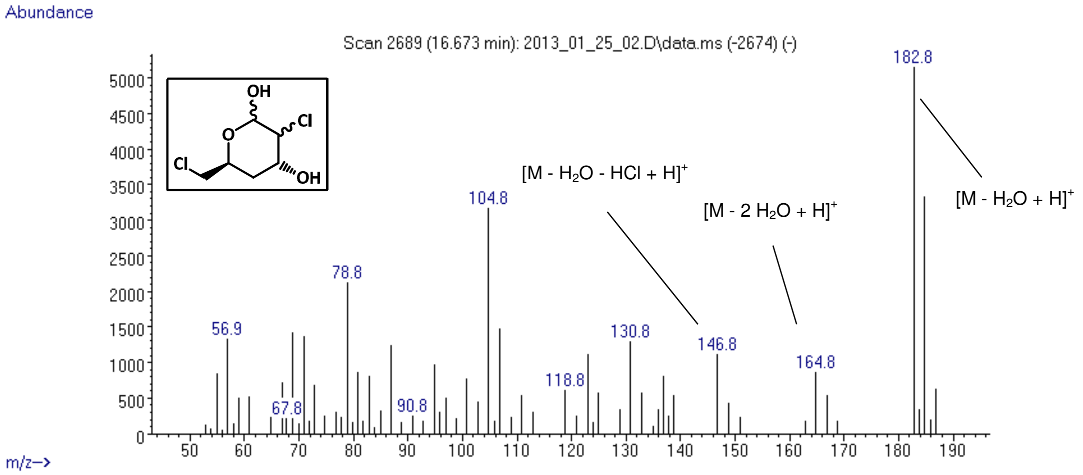


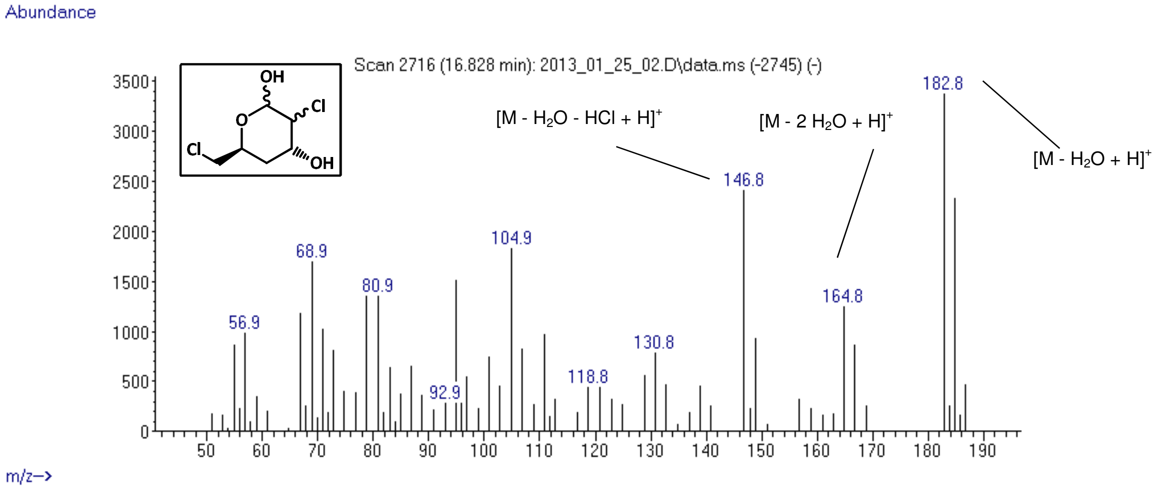


**Figure S9 part 2: GC-MS spectra indicating the structure of 19.**
