# Supplementary material for: A Highly Productive, Whole-Cell DERA Chemoenzymatic Process for Production of Key Lactonized Side-Chain Intermediates in Statin Synthesis
Source: PLoS One. 2013 May 7;8(5):e62250. doi: 10.1371/journal.pone.0062250 (PMC3647077; doi:10.1371/journal.pone.0062250)
Supplement: Information S9 — A fed-batch process keeping 2g concentration near dynamic zero. (PDF) [file pone.0062250.s009.pdf]

Matej Ošlaj,[a] Jérôme Cluzeau,[b] Damir Orkić,[b] Gregor Kopitar,[a] Peter Mrak[a] and Zdenko Časar[b,c]**

Supporting information S9. A fed-batch process keeping 2g concentration near dynamic zero.

Reactions using (in total) 400 mmol L^{-1} of **2g** and 840 mmol L^{-1} of **1** were set up, having slightly different feeding profile for acetaldehyde compared to the process described in the main article Figure 7. Whole-cell catalyst (*E. coli* BL21 (DE3) pET30/*deoC*) with 217 kRFU s^{-1} g^{-1} DERA specific activity and 182 g L^{-1} WCW was used. The faster addition of the acetaldehyde in the first 30 minutes of the reaction results in low dynamic concentrations of **2g**, which are well below the dynamic concentrations of **8g** and of acetaldehyde. Despite this concentration differences, the rate of accumulation of **8g** is still higher compared to the rate of formation of **3g** (Figure SP10).

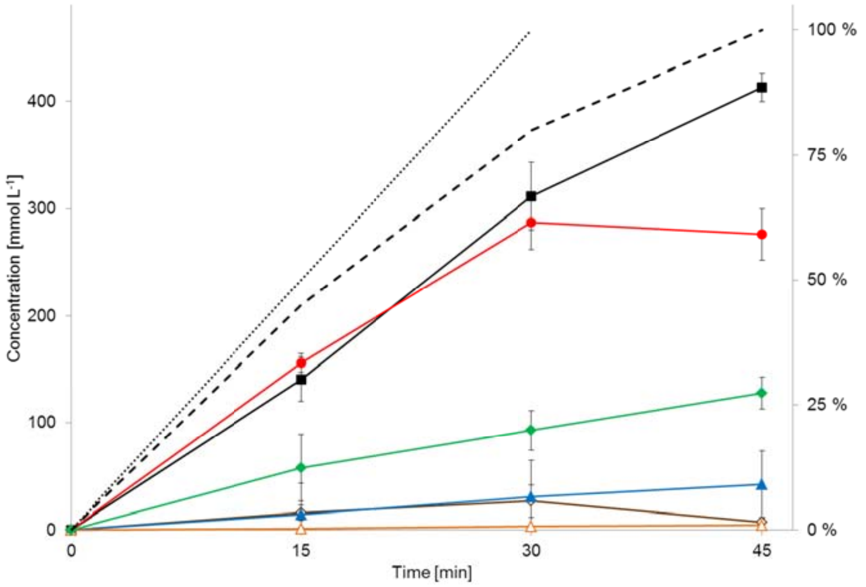

Figure S10. Time course of DERA whole-cell-catalyzed, fed-batch reactions yielding 3g. Reaction species data from 3 independent reactions using (in total) 400 mmol L^{-1} of **2g** and 840 mmol L^{-1} of **1** are shown. Whole-cell catalyst (*E. coli* BL21 (DE3) pET30/*deoC* high-density culture) with 217 kRFU s^{-1} g^{-1} DERA specific activity and 182 g L^{-1} WCW was used. Results are given as molar concentrations obtained from GC-FID analysis: **1** (■, black), **3a** (▲, blue) **3g** (♦, green), **8g** (●, red), **10g** (Δ, orange), **2g** (◊, brown). Secondary vertical axis shows in %, cumulative feed of **2g** (dotted line), and cumulative feed of **1** (dashed line). All reaction species except **3a** and **3g** are found and measured in their aldehyde forms at the GC-FID analysis conditions. At reaction conditions, the measured quantity of a specific reaction species is divided between the corresponding aldehyde, hydrate and acetal /hemiacetal equilibrium forms.
