# Supplementary material for: A Highly Productive, Whole-Cell DERA Chemoenzymatic Process for Production of Key Lactonized Side-Chain Intermediates in Statin Synthesis
Source: PLoS One. 2013 May 7;8(5):e62250. doi: 10.1371/journal.pone.0062250 (PMC3647077; doi:10.1371/journal.pone.0062250)
Supplement: Information S10 — Accumulation of acetic acid in reactions with 2g. (PDF) [file pone.0062250.s010.pdf]

Matej Ošlaj,[a] Jérôme Cluzeau,[b] Damir Orkić,[b] Gregor Kopitar,[a] Peter Mrak[a] and Zdenko Časar[b,c]**

Supporting information S10. Accumulation of acetic acid in reactions with 2g

Comparison of the high-productivity, fed-batch, whole-cell process using **2g** or **2b** as one of the substrates, with focus to the minor reaction species is shown below. Data from the same experiments as shown in Figure 8 of the main article are depicted in Figure S11. A small amount of acetic acid was found accumulating in the reaction with **2g**, but was not detected in the reaction with **2b**.

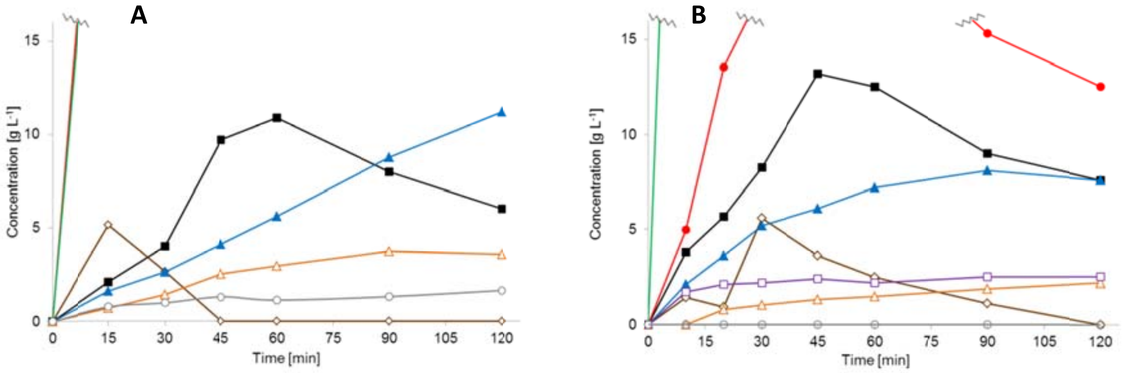

Figure S11. Comparison of time course of DERA whole-cell-catalyzed, fed-batch reactions yielding 3b or 3g. Whole-cell catalyst (E. coli BL21 (DE3) pET30/deoC high-density culture) with 247 kRFU s^{-1} g^{-1} DERA specific activity and 215 g L^{-1} WCW was used. Results are given as mass concentrations obtained from GC-FID analysis. The measured quantity of a particular compound, with the exception of the stable 6-ring hemiacetals (**3**), represents the sum of the corresponding equilibrium forms (hydrate, aldehyde and acetal/hemiacetal) which exist under the reaction conditions. Charts are focused to show behavior of the minor reaction species in more detail. **A**: Reaction species data from reaction using (in total) 700 mmol L^{-1} of **2g** and 1540 mmol L^{-1} of **1** are shown. **1** (■, black), **3a** (▲, blue) **3g** (♦, green), **8g** (●, red), **10g** (Δ, orange), **2g** (◊, brown), acetic acid (○, grey). **B**: Reaction species data from reaction using (in total) 700 mmol L^{-1} of **2b** and 1540 mmol L^{-1} of **1** are shown. **1** (■, black), **3a** (▲, blue) **3b** (♦, green), **8b** (●, red), **10b** (Δ, orange), **2b** (◊, brown), acetic acid (○, grey), 2,6-chloro-2,4-dideoxyhexose (□, purple).
